# Thyroid Hormone Enhances Angiogenesis and the Warburg Effect in Squamous Cell Carcinomas

**DOI:** 10.3390/cancers13112743

**Published:** 2021-06-01

**Authors:** Caterina Miro, Annarita Nappi, Annunziata Gaetana Cicatiello, Emery Di Cicco, Serena Sagliocchi, Melania Murolo, Valentina Belli, Teresa Troiani, Sandra Albanese, Sara Amiranda, Ann Marie Zavacki, Mariano Stornaiuolo, Marcello Mancini, Domenico Salvatore, Monica Dentice

**Affiliations:** 1 Department of Clinical Medicine and Surgery, University of Naples Federico II, 80131 Naples, Italy; caterina.miro@unina.it (C.M.); annarita_nappi@libero.it (A.N.); Annunziatagaetana.cicatiello2@unina.it (A.G.C.); emery.dicicco@unina.it (E.D.C.); serena.sagliocchi@unina.it (S.S.); Mel.murolo@studenti.unina.it (M.M.); 2 Laboratorio di Oncologia Molecolare, Dipartimento di Medicina di Precisione, University of Campania Luigi Vanvitelli, 81100 Caserta, Italy; valentina.belli@hotmail.com (V.B.); teresa.troiani@unicampania.it (T.T.); 3 Institute of Biostructures and Bioimaging of the National Research Council, 80131 Naples, Italy; sandra.albanese@ibb.cnr.it (S.A.); direttore@ibb.cnr.it (M.M.); 4 Department of Molecular Medicine and Medical Biotechnology, University of Naples Federico II, 80131 Naples, Italy; sara.amiranda@unina.it; 5 CEINGE–Biotecnologie Avanzate Scarl, 80131 Naples, Italy; domsalva@unina.it; 6 Harvard Medical School, Brigham and Women’s Hospital, Boston, MA 01451, USA; azavacki@rics.bwh.harvard.edu; 7 Department of Pharmacy, University of Naples Federico II, 80131 Naples, Italy; mariano.stornaiuolo@unina.it; 8 Department of Public Health, University of Naples Federico II, 80131 Naples, Italy

**Keywords:** thyroid hormones, deiodinases, angiogenesis, squamous cell carcinoma

## Abstract

**Simple Summary:**

Cancer cells rewire their metabolism to promote growth, survival, proliferation, and long-term maintenance. Aerobic glycolysis is a prominent trait of many cancers; contextually, glutamine addiction, enhanced glucose uptake and aerobic glycolysis sustain the metabolic needs of rapidly proliferating cancer cells. Thyroid hormone (TH) is a positive regulator of tumor progression and metastatic conversion of squamous cell carcinoma (SCC). Accordingly, overexpression of the TH activating enzyme, D2, is associated with metastatic SCC. The aim of our study was to assess the ability of TH and its activating enzyme in promoting key tracts of cancer progression such as angiogenesis, response to hypoxia and metabolic adaptation. By performing in vivo and in vitro studies, we demonstrate that TH induces VEGF-A in cancer cells and fosters aerobic glycolysis inducing pro-glycolytic mediators, thus implying that TH signal attenuation represents a therapeutic tool to contrast tumor angiogenesis and tumor progression.

**Abstract:**

Cancer angiogenesis is required to support energetic demand and metabolic stress, particularly during conditions of hypoxia. Coupled to neo-vasculogenesis, cancer cells rewire metabolic programs to sustain growth, survival and long-term maintenance. Thyroid hormone (TH) signaling regulates growth and differentiation in a variety of cell types and tissues, thus modulating hyper proliferative processes such as cancer. Herein, we report that TH coordinates a global program of metabolic reprogramming and induces angiogenesis through up-regulation of the *VEGF-A* gene, which results in the enhanced proliferation of tumor endothelial cells. In vivo conditional depletion of the TH activating enzyme in a mouse model of cutaneous squamous cell carcinoma (SCC) reduces the concentration of TH in the tumoral cells and results in impaired VEGF-A production and attenuated angiogenesis. In addition, we found that TH induces the expression of the glycolytic genes and fosters lactate production, which are key traits of the Warburg effect. Taken together, our results reveal a *TH–VEGF-A–HIF1α* regulatory axis leading to enhanced angiogenesis and glycolytic flux, which may represent a target for SCC therapy.

## 1. Introduction

Cancer angiogenesis, stimulated by mutual interactions between tumor cells and endothelial cells (ECs), sustains cancer cell progression by supplying nutrients and oxygen. The expansion of the vascular network is essential for tumor growth and metastatization, providing structural support to tumor needs during cancer progression [1,2,3,4]. One of the primary stimuli for tumor angiogenesis is evoked by hypoxia, a consistent decrease in O_2_ tension, and nutrient concentrations. Hypoxia is a frequent feature of solid tumors, driven by the activation of the hypoxia-inducible transcription factors (HIF) that, in turn, lead to the expression of different target genes [5,6,7,8]. Pathologically activated angiogenesis is often associated with metabolic reprogramming characterized by an increase in glycolytic fluxes and lactate secretion, even in normoxic condition, a process called the Warburg effect [5,6,7,8]. Thyroid hormone (TH) is a critical mediator of development and energy metabolism of several tissues in vertebrates. TH is produced by the thyroid gland in form of T4 (pro-hormone) and T3 (active hormone), enters the circulation, and reaches target tissues, thereby regulating the expression of its target genes [9]. The biological effects of TH in peripheral tissues are mediated by TH transporters, deiodinases and TH receptors, regardless of relatively constant serum levels [9]. The enzymatic activity of three iodothyronine deiodinases represents the major classical pathway of local TH metabolism and a potent mechanism for pre-receptor regulation of TH action at the cellular level. In detail, the type 1 or 2 selenoproteins catalyze the conversion of the inactive pro-hormone, T4, to the active form, T3; conversely, D3 terminates TH action by converting both T3 and T4 to inactive metabolites [10]. Different studies have demonstrated that TH deregulation leads to the onset of different human tumors, characterized by thyroid hormone receptors and deiodinases alterations [11,12]. Interestingly, TH affects various oncogenic and tumor suppressor pathways that, in turn, alter the balanced expression of D2 and/or D3, thus resulting in a feedback loop that amplifies or attenuates tumor growth [13].

One of the first pieces of evidence linking the role of TH with neoplastic transformation was highlighted by the finding that TRα1 is the cellular counterpart of the oncogene *v-ErbA*, carried by the avian erythroblastosis virus (AEV) [14]. Moreover, it has been demonstrated that regulation of the intracellular TH levels affects tumor formation by controlling different oncogenic signaling pathways [11,12].

We previously evaluated the role of thyroid hormone (TH) and its activating enzyme, type 2 deiodinase, D2, in cancer progression, and found that TH is a critical regulator of Squamous Cell Carcinoma (SCC) tumorigenesis and that TH activation by D2 enhances the invasiveness of SCCs and their tendency to metastasize [15,16,17]. Cutaneous squamous cell carcinoma is the second most frequent cancer in humans and can undergo metastatic formation [18]. Animal models for skin squamous tumors are ideal for the study of cancer initiation and progression [19]. The most widely used mouse SCC cancer model is the multistage chemically induced skin carcinogenesis model [20]. We also found that D2 expression is up-regulated in SCC tumorigenesis at the papilloma stage and that its expression is associated with the most aggressive stages of mouse and human SCC tumorigenesis [16]. Accordingly, clinical observations revealed that high-D2 tumors are associated with a lower survival rate and enhanced relapse frequency. Finally, we demonstrated that the molecular mechanism by which the D2-mediated TH activation fosters cancer progression is the up-regulation of the epithelial–mesenchymal switch of SCC cells through the induction of ZEB1 and repression of E-cadherin [16].

Here, we reveal that TH sustains the survival and metabolic demands of SCC cells by other, as yet unknown, means. In fact, our data show that TH positively regulates the transcription of *VEGF-A* and the expression of the hypoxia-induced factor HIF1, which are essential traits of cancer cells [21]. Linked to the tumor-promoting effects of TH is the ability to induce the metabolic reprogramming of SCC cells. By using an inducible cell line in which the *DIO2* gene can be overexpressed upon doxycycline (DOX, 2 μg/mL, Clontech, Mountain View, CA, USA) administration [22], we show that TH fosters the expression of key mediators of the Warburg effect of cancer cells, namely, GLUT1, PKM2 and LDHA.

The close link between angiogenesis and the Warburg effect is demonstrated by the reciprocal interplay between cancer cells that secrete lactate and acidify the extracellular medium, thereby enhancing the activation of endothelial cells and inducing the neo-vasculogenesis of SCC tumors. On the other hand, increased oxygen and nutrient supply to cancer cells is an absolute requisite for the metabolic requirements of cancer cells. Thus, our data demonstrate that TH and its modulating enzyme D2 are critical determinants of SCC tumorigenesis and the metabolic adaptation of cancer cells. Furthermore, our data support a role of TH in the regulation of vascular biology and response to hypoxia, which, together, are the main tracts of survival of cancer cells during tumor growth and metastatization. The parallel ability of TH in fostering metabolic adaptations of cancer cells that enhance glycolytic fluxes reinforces the paradigm by which TH constitutes a remarkable endocrine regulator of tumor progression. These proof-of-principles indicate that TH and the activating enzyme, D2, are ideal candidate targets for the pharmacological therapy of tumor growth.

## 2. Materials and Methods

### 2.1. Cell Cultures

Human coronary artery endothelial cells (HCAECs) were cultured in Endothelial Cell Growth Medium v2 (EGMv2, Cell Applications, Weathers Pl, San Diego, CA, USA, Cat No. 213–500). B16-OVA is an ovalbumin-transfected clone derived from the murine melanoma cell line B16-F10. We cultured B16-OVA cells in Dulbecco’s modified Eagle medium (Gibco, Thermofisher Scientific, Waltham, MA, USA) supplemented with 10% Fetal Bovine Serum (FBS) (Gibco, Thermofisher Scientific), 2 mM L-Glutamine (Gibco, Thermofisher Scientific), 50 i.u. (Gibco, Thermofisher Scientific), and 50 μg/mL streptomycin (Gibco, Thermofisher Scientific). SCC13 cells were derived from a skin SCC [23]. SCC13 cells, SCC D2KO cells and pTRE-D2 SCC13 cells were cultured in keratinocyte-SFM (KSFM 1×, Gibco, Thermofisher Scientific, cod. 17005042) serum-free medium [+] L-Glu (Gibco, Thermofisher Scientific) with bovine pituitary extract (30 μg/mL) and human recombinant epidermal growth factor (EGF) protein (0.24 ng/mL). pTRE-D2 SCC13 cells were selected using 100 μg/mL geneticin (G418) (Biowest, Nuaillé, France) and 0.2 μg/mL puromycin (Invitrogen, Carlsbad, CA, USA). Type 2 deiodinase is switched on by the Tet-On expression system (pTRE-D2 SCC13 cells) activated by doxycycline (2 μg/mL, Clontech, Mountain View, CA, USA). In all the experiments in which thyroid hormone was applied to cells, we used a combination of T3 and T4 (Sigma-Aldrich, St. Louis, MI, USA) (10 nM each, indicated throughout the text as “TH”), thus resembling the physiological exposure of cells to both the active hormone (T3) and its pro-hormone (T4). In experiments in which TH was removed from the serum, TH-depletion was achieved by FBS charcoal absorption [24]. EC cells in some experiments were treated with Bevacizumab (Zirabev, Pfizer, New York, NY, USA, 25 mg/mL) at a final concentration of 500 ng/mL for 24 h. For co-culture experiments, in order to separate HCAEC and SCC13 WT or D2KO cells in different compartments, we utilized the transwell permeable supports with a 0.4 μm polycarbonate membrane (Corning, Tewksbury, MA, USA, cod. 3401).

A total of 50,000 HCAECs were grown in 500 μL EGMv2 in 12-well plates. An equivalent number of SCC13 WT or D2KO cells were plated into the top chamber of a transwell insert, placed on a 12-well plate containing the HCAECs (Figure S1A).

### 2.2. DIO2 Targeted Mutagenesis

We performed the targeted mutagenesis of *DIO2* gene in SCC13 cells by the CRISPR/Cas9 system from Santa Cruz Biotechnology (Santa Cruz, Dallas, TX, USA). CRISPR/Cas9 control plasmid was used as a control, as previously described [25]. We executed fluorescence-activated cell sorting (FACS) for green fluorescent protein (GFP) expression to isolate positive cells 24 h after transfection.

We assessed *DIO2* exon 1 inserted mutations by sequencing. The experiments in D2KO cells were conducted in three different D2KO clones to avoid off-target effects.

### 2.3. Constructs and Transfections

The set of vectors for tetracycline-inducible transgene expression, constituted by the response pTRE3G, the regulator pCMV-Tet3G and the pTRE3G-Luc control plasmids, was purchased from Clontech Laboratories (Clontech, Mountain View, CA, USA) and their use and modifications were achieved as previously described [22].

### 2.4. Conditional Dio2 Expression in SCC13 Cells

Transfection experiments were performed in SCC13 cells as previously described to obtain conditional *Dio2* gene expression [22] (Figure S1A,B). Clones’ selection was performed in media with 100 μg/mL G418 (Biowest) and 0.2 μg/mL puromycin (Invitrogen), yielding a total of 80 clones. Cells were collected after 48 h of DOX induction and Western blot using a monoclonal anti-Flag M2 antibody (Sigma-Aldrich, cod. F3165, 1:1000), to detect the D2-Flag. Two different clones (both derived from the clone 19) showed a strong induction of D2 expression following DOX treatment and were selected for further studies (Figure S1A,B). Two clones with the highest fold induction of the D2 protein (clone 19_8 and 19_21) were selected for propagation and further testing.

### 2.5. Protein Extraction from Skin and Western Blot Analysis

Mice dorsal skin was resuspended in 800 μL of lysis buffer (0.125 M Tris pH 8.6; 3% SDS, protease inhibitors including phenylmethylsulfony fluoride (PMSF) (Sigma-Aldrich, cod. P7626) and phosphatase inhibitors (Sigma-Aldrich, cod. P8340)) and then homogenized with Tissue Lyser (QIAGEN, Stockach, Germany). Total protein extracts were run on a 10% SDS-PAGE gel and transferred onto an Immobilon-P transfer membrane (Millipore, Burlington, MA, USA). The following primary antibodies and dilutions were used: VEGF-A (abcam, Cambridge, UK, cod. ab52917, 1:500), CD31 (abcam, cod. ab28364, 1:500), αFlag M2 (F3165 Sigma Aldrich, 1:1000), HIF1α (BD Biosciences, San Jose, CA, USA, 610958, 1:500), Tubulin (T8203, Sigma-Aldrich, 1:10,000), GLUT-1 (ab652, 1:1000), PKM1 ab116271, 1:1000), PKM2 (abcam, cod. ab137791, 1:1000), LDHA (sc-130327, 1:1000), LDHB (abcam, cod. ab85319, 1:1000), Enolase-1 (Cell Signaling #3810, Boston, MA, USA, 1:1000), Aldolase A (Cell Signaling #8060, 1:1000). We used anti-mouse IgG-horseradish peroxidase (HRP) (Biorad, Hercules, CA, USA, cod. 1706516) and anti-rabbit IgG-HRP (Biorad, cod. 1706515) secondary antibodies, detected by chemiluminescence (Millipore, Burlington, MA, USA, cat. WBKLS0500). Anti-Tubulin specific antibodies (Sigma-Aldrich, cod. T8203) were loaded as controls. All Western blots were run in triplicate, and bands were quantified with Image J software (NIH Image, Bethesda, MD, USA). The full western blots can be found at Figure S10.

### 2.6. Real-Time PCR

Messenger RNAs were obtained through resuspension of the samples with Trizol reagent (Life Technologies Ltd., Carlsbad, CA, USA). Complementary DNAs were prepared with Vilo reverse transcriptase (Invitrogen, cod. 11755), as indicated by the manufacturer. The cDNAs were amplified by real-time in a CFX Connect Real-Time PCR Detection System (BioRad) with the fluorescent double-stranded DNA-binding dye SYBR Green (Biorad, cod. 1708882). Each gene was tested using specific primers developed to amplify at the same cycling conditions (95 °C for 10 min followed by 40 cycles at 95 °C for 15 s and 60 °C for 1 min), thereby giving products of comparable sizes (about 200 bp for each amplification). We designed primers that matched to exon–exon junctions and digested RNA with DNAse in order to impair genomic contamination. Primer sequences are indicated in the Table 1. For each reaction, standard curves for reference genes were constructed based on six fourfold serial dilutions of cDNA. Cyclophilin A was used as the control housekeeping gene to relate the expression of genes of interest. The results, expressed as N-fold differences in target gene expression, were determined as follows: N*target = 2^(ΔCt sample−ΔCt calibrator)^.

### 2.7. Chromatin Immunoprecipitation (ChIP) Assay

SCC13 cells were treated with T3 and T4 (10 nM) for 48 h. Briefly, 2 × 10^6^ cells were fixed with 1% formaldehyde (Sigma-Aldrich, cod. F8775) for 10 min at 37 °C in growth medium. The reaction was extinguished by adding glycine to a final concentration of 0.125 M. We resuspended fixed cells in 1 mL of lysis enriched with protease inhibitors (200 mM PMSF, Sigma-Aldrich, cod. P7626), 1 μg/mL aprotinin (Sigma-Aldrich, cod. A6279). The lysis products were sonicated to achieve DNA fragments of 200–1000 bp. After sonication, samples were centrifuged, and the chromatin present in the supernatant was diluted with 10 volumes of dilution buffer. Sonicated samples were centrifuged, and the soluble chromatin was diluted 10-fold in dilution buffer and used directly for ChIP assays. An aliquot (1/10) of fragmented chromatin was further processed with proteinase K (Invitrogen, cod. 25530); consequently, DNA was obtained by phenol/chloroform extraction in order to evaluate the concentration and sonication efficiency (“input DNA”). The sheared chromatin was pre-cleared for 2 h with 1 μg of non-immune IgG (Sigma-Aldrich) and 30 μL of Protein G Plus/ Protein A Agarose suspension (Sigma-Aldrich, GE Healthcare, cod. GE170780) saturated with salmon sperm (1 mg/mL). Precleared chromatin was partitioned and left at 4 °C for 16 h with 1 μg of anti-TRα (Abcam, cod. ab2743). Bound DNA-protein complexes, washed five times, were eluted by incubation with 1% sodium dodecyl sulfate (Sigma-Aldrich, cod. L3771)-0.1 M NaHCO_3_ elution buffer. Formaldehyde cross-links were reversed by exposition to 200 mM NaCl (Sigma-Aldrich, cod. 71376) at 65 °C. Samples were extracted twice with phenol–chloroform and washed with ethanol. DNA fragments were precipitated, resuspended in 50 μL H_2_O, and tested with real-time PCRs.

### 2.8. Transwell Migration Assay

A total of 1.5 × 10^5^ cells/mL of HCAECs were added to the upper chamber of a 12-well plate transwell insert with 8.0 µm polycarbonate membrane (Corning, Tewksbury, MA, USA, cod. 353182). The lower chamber was filled with EGMv2 supplemented with 20% conditional medium (CM) collected from either WT or D2KO SCC13 cells. After 3 h, the migration ability was evaluated with DAPI live staining of HCAEC migrated in the lower chamber and then imaged at 20× using a Leica DMi8 microscope (Leica Microsystems, Wetzlar, Germany). The cells were randomly counted (Figure S2E).

### 2.9. Hypoxia Experiments

Hypoxic exposure was performed using a modular hypoxia chamber (Stemcell technologies, Vancouver, Canada, cod. 27310) (1% O_2_, 5% CO_2_, with N_2_ balance at 37 °C) or with the use of 100 μM CoCl_2_ (Sigma-Aldrich, cod. 15862) [26,27].

### 2.10. Seahorse

Seahorse XF-96 extracellular flux analyzer (Seahorse Biosciences, Billerica, MA, USA) was used to detect the extracellular acidification rate (ECAR) due to glycolytic activity. The day before the assay, sensor cartridges were hydrated with calibration buffer and positioned in a 37 °C incubator without CO_2_ (Thermo Electron Corporation, Waltham, MA, USA). Cells were incubated with Seahorse XF Base Medium (Agilent Technologies, West, Cedar Creek, TX, USA, cod. 103576-) (pH 7.4) composed of 1 mM pyruvate (Agilent Technologies, cod. 103578), 2 mM glutamine (Agilent Technologies, cod. 103579), 10 mM glucose (Agilent Technologies, cod. 103577), and then left in a 37 °C incubator without CO_2_ for 1 h before the analysis, using the Glycolytic Rate assay (Agilent Technologies, cod. 103344). For the measurement, 0.5 μM Rotenone/Antimycin A and then 50 mM 2-Deoxyglucose were added to the cells, according to the manufacturer’s instructions. Seahorse data, normalized to cell counts, were evaluated using Wave, software for XFp Analyzer (Agilent Technologies) and Prism (GraphPad software, San Diego, CA, USA) software was used to generate statistics and graphical representations. ATP levels were calculated using the ATPlite 1 step kit (PerkinElmer, Waltham, MA, USA, cod. 6016736) according to the manufacturer’s instructions.

### 2.11. Animals, Histology and Immunostaining

sD2KO (K14-CreER; D2fl/fl) and sD3KO (K14-CreER;D3fl/fl) mice were obtained by crossing the keratinocyte-specific conditional K14-CreER mouse [28] with D3fl/fl [29] or D2fl/fl murine models [30]. The C57BL/6 mouse strain was used in both cases. Depletion was induced by the administration of 10 mg of tamoxifen at different time points, reported in each experiment. The generation of D2-3xFlag has already been described [31]. Skin lesions were collected at different time points, following tamoxifen (Sigma-Aldrich St. cod. T5648) administration and DMBA-TPA (Sigma-Aldrich, cod. D3254 and cod. P8139) treatment, in which the “initiation” step consists of treatment with a low dose of the mutagen 9,10-dimethyl-1,2-benzanthracene (DMBA) (Sigma-Aldrich, cod. D3254), and the “promotion” stage consists of treatment with 12-O-tetradecanoyl phorbol-13-acetate (TPA) (Sigma-Aldrich, cod. D3254 and cod. P8139). This procedure induces epidermal proliferation and causes the formation of benign tumors (papillomas) and their progression to invasive SCC. Hyperthyroid mice were obtained by treating 12-week-old C57BL/6 male mice (The Jackson Laboratory, Bar Harbor, ME, USA) with T3 (1 mg/mL) and T4 (4 mg/mL) in drinking water for three weeks. For immunofluorescence and histology, dorsal skin from sD2KO, sD3KO, D2-3xFlag and control mice was embedded in paraffin, cut into 7 μm sections, and H&E-stained, as previously described [16]. The following primary antibodies and dilutions were used: VEGF-A (ab52917, 1:200), CD31 (ab28364, 1:500), VEGF Receptor 2 (Cell signaling 2479, 1:200), Phospho-VEGF Receptor 2 (Tyr 1175) (Cell Signaling 3770, 1:200). Images were acquired with a Leica DMi8 microscope (Leica microsystems, Wetzlar, Germany) and the Leica application suite LASX Imaging software (Leica microsystems).

For FACS analysis of CD31-positive cells, papillomas and carcinomas arising from DMBA/TPA D2KO and D2WT mice were digested in collagenase I (Sigma-Aldrich, cod. C0130) for 2 h at 37 °C on a rocking plate. After tumor digestion, cells were filtered through a 70 μm cell strainer. For immunostaining, APC-anti-mouse CD31 (BD561814) and APC-anti mouse CD45 (BD553081) antibodies were incubated for 1 h. Fluorescence-activated cell analysis was performed using FACS Canto2 software (FACS Canto2, Becton Dickinson, ITALIA S.p.A). 

All animal studies were conducted in accordance with the guidelines of the Italian Ministry of Health and were approved by the Institutional Animal Care and Use Committee (IACUC, n. 167/2015-PR and n. 354/2019-PR).

### 2.12. DMBA/TPA Carcinogenesis

Skin lesions were obtained by treating the dorsal skin of 2-month-old mice with a single dose (100 μL, 1 mg/mL) of the carcinogen 7,12-dimethylbenz[a]anthracene (DMBA) (Sigma-Aldrich, cod. D3254), resuspended in propanone (VWR Chemicals, Radnor, PA, USA, cod. 20065.327) followed by multiple applications of the tumor promoter 12-O-tetradecanoylphorbol-13-acetate, TPA (150 μL, 100 μM) Sigma-Aldrich, cod. P8139 [32]. Experiments with D3fl/fl mice were performed using 15 D3WT mice and 15 D3KO mice. Experiments in D2fl/fl mice were performed using a total of 15 D2WT mice and 15 D2KO mice. Experiments in D2-Flag mice were performed using 8 mice. In all the cohorts, there were approximately as many female as male mice [33].

### 2.13. Non-Invasive High Frequency Ultrasound of Xenograft and Lymph Nodes

A high-frequency ultrasound (HFUS) system (VEVO 2100, FUJIFILM VisualSonics, Inc., Toronto, ON, Canada), mounted with 40 MHz and 50 MHz transducers (MS 550 D, MS770 FUJIFILM VisualSonics), was used to evaluate both tumor and lymph node morphology and vascularization. The HFUS evaluations were conducted in anesthetized mice (2% isoflurane in 100% oxygen at 0.8 L/min). Each mouse was placed in prone recumbency on a dedicated heated platform (VEVO Imaging Station 2, FUJIFILM VisualSonics), and accurately shaved to remove hairs from the region of interest. Temperature was maintained using a heating lamp and monitored using a rectal probe.

Brightness (B-) mode and color-doppler mode images were obtained for each tumor and lymph nodes in two orthogonal planes, i.e., the trans-axial and the sagittal planes. After bidimensional acquisitions, the transducer was placed in a sagittal orientation and a stepper motor was used to acquire a three-dimensional data set (resolution of 0.018 × 0.018 × 0.076 mm^3^) with power Doppler mode, across the whole tumor lesion, using respiratory gating: total scan time was approximately 5 min. Regions of interest were drawn by hand around the tumor borders on every second acquired ultrasound two-dimensional slice. Then, VEVO 2100 software (FUJIFILM VisualSonics) automatically determined the percentage of vascularization of the tumor volume obtained by segmentation.

### 2.14. Angiogenesis-Related Antibody Array

The Mouse Angiogenesis Array Kit was purchased from R&D Systems (Catalog number ARY015, Minneapolis, MN, USA). Tissue samples were excised and homogenized in PBS with protease inhibitors (10 μg/mL Aprotinin (Sigma-Aldrich, cod. A6279), 10 μg/mL Leupeptin (Tocris, Bristol, UK, cod. 1167), and 10 μg/mL Pepstatin (Tocris, cod. 1190). Total protein levels were quantified, and 300 mg of total mouse protein samples was analyzed with the Mouse Angiogenesis Array Kit (R&D Systems, Minneapolis, MN, USA, cod. ARY015). The procedure was performed according to the manufacturer’s instructions.

The angiogenesis antibody membrane was incubated with 2 mL of array blocking buffer for 30 min on a rocking platform. After incubation, the antibody membrane was washed twice with the array wash buffer, and 1.5 mL of each tissue sample were added to the well. The membrane was incubated overnight at 4 °C on a rocking platform shaker. The membrane was then washed twice with 20 mL of 1× Wash Buffer. Then, 2 mL of diluted streptavidin–horseradish peroxidase solution was added into each well of a 4-well multi-dish containing the membrane and incubated for 30 min at room temperature on an orbital shaker. After three washes with array wash buffer I, a chemiluminescent substrate ECL kit (R&D Systems, Chemi reagent 1 cod. 894287, Chemi reagent 2 cod. 894288), supplied with the kit, was used to obtain detailed images of the array. Image J software was used to quantify the arrays.

### 2.15. Statistics

The results are reported as means ± SD throughout the study. For experiments in which we compared two different conditions, Student’s two-tailed t tests were applied. For the experiments in which multiple conditions were compared, we used the one-way ANOVA test. All the data were obtained by performing at least three independent experiments, each in duplicate or in triplicate, and the statistical analysis was applied to the independent triplicates. Relative mRNA levels (in which the control sample was arbitrarily set as 1) are reported as results of real-time PCR in which the expression of cyclophilin A served as the housekeeping gene. In all experiments, differences were considered significant when *p* was less than 0.05. Asterisks indicate significance at * *p* < 0.05, ** *p* < 0.01, and *** *p* < 0.001 throughout.

## 3. Results

### 3.1. Thyroid Hormone Induces VEGF-A Expression in SCC Cells and Promotes VEGF-A-Dependent Angiogenesis

**Figure 1:**
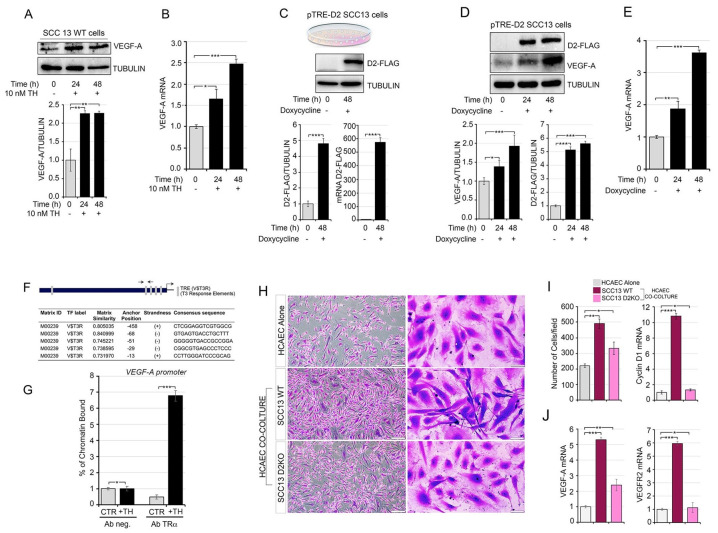
TH promotes VEGF-A secretion in SCC cancer cells and increases endothelial cell proliferation. (**A**) SCC13 cells were treated with 10 nM TH for 24 and 48 h. Total protein lysates were used for Western blot analysis of VEGF-A expression. Quantification of the VEGF-A protein levels versus tubulin levels is represented by a diagram (bottom). (**B**) mRNA levels of VEGF-A in SCC cells were treated as in (**A**,**C**). Schematic representation of the doxycycline treatment of pTRE-D2 SCC13 cells. D2-FLAG protein and D2 mRNA was evaluated by Western blot analysis and real-time PCR in pTRE-D2 SCC13 cells ± 2 μg/mL doxycycline for 48 h (bottom). (**D**) Western blot analysis of VEGF-A and D2-FLAG expression was performed in pTRE-D2 SCC13 cells after doxycycline treatment for 24 and 48 h. Quantifications of the VEGF-A and D2-FLAG protein versus Tubulin levels are represented by diagrams (bottom). (**E**) mRNA levels of VEGF-A in pTRE-D2 SCC13 cells after D2 induction were measured by real-time PCR analysis. (**F**) Schematic representation of the human VEGF-A promoter and the potential TR binding sites identified by in silico analysis of the VEGF-A promoter and indicated in gray. The sequence of five consensus TR binding sites identified upstream of the transcriptional start site (TSS) is shown in the bottom panel. (**G**) Chromatin immunoprecipitation of TR binding to the VEGF-A promoter was performed in SCC13 cells treated or not with TH for 48 h. (**H**) The EC proliferation rate was evaluated by transwell assay; the images show the HCAEC cells labelled with crystal violet after 5 days of co-culture with SCC WT, SCC D2KO or alone. Scale bars represent 50 μm. (**I**) Quantitative results (number of cells) of HCAEC transwell assay (left) and mRNA expression level of Cyclin D1 in HCAEC cells cultured as indicated in H (right). (**J**) mRNA expression of VEGF-A and VEGFR2 was measured in HCAEC cells cultured as indicated in H. Each experiment was done in triplicate. Results are expressed as mean ± SD. * *p* < 0.05, ** *p* < 0.01, *** *p* < 0.001.

Recently, *VEGF-A* was found to be a positive TH target gene in C2C12 muscle cells, in which TH promotes VEGF-A secretion and VEGF-A-mediated stimulation of muscular endothelial cells [34]. Given the tumor-promoting effects of TH in SCC tumor progression [16], we asked if, similar to skeletal muscle, TH induces VEGF-A in SCC cells. First, we evaluated the expression of VEGF-A in the human skin squamous cell carcinoma cell line SCC13 after TH treatment. We observed that VEGF-A protein and mRNA expression levels were higher in TH-treated cells than in control cells (Figure 1A,B). To assess whether TH intracellular activation via the D2 enzyme was also involved in VEGF-A up-regulation, we generated an SCC inducible cell line in which the D2 enzyme was reversibly turned on by the Tet-On expression system (pTRE-D2 SCC13 cells) activated by DOX treatment (Figure 1C and Figure S1A,B). In this cell model, DOX-induced D2 expression caused VEGF-A up-regulation at both the protein and mRNA level (Figure 1D,E). Accordingly, inhibition of D2 activity via rT3 treatment (which acts as a competitive inhibitor of D2 [35]), reduced the basal levels of VEGF-A, thus proving the physiological relevance of D2 in the VEGF-A expression (Figure S2A,B).

Next, we assessed if *VEGF-A* is a direct target of TH in SCC cells by performing a chromatin immunoprecipitation (ChIP) assay, which confirmed that the TH TRα receptor physically binds *VEGF-A* in the human skin squamous cell carcinoma cells (SCC 13) (Figure 1F,G).

VEGF-A is a soluble factor produced by tumoral cells to stimulate angiogenesis in the tumor microenvironment; therefore, we investigated the role of TH-induced VEGF-A production by SCC cancer cells on the EC proliferation. In detail, to test the hypothesis that TH promotes VEGF-A secretion in SCC cells, thereby affecting EC proliferation, we performed a co-culture experiment with human coronary artery endothelial cells (HCAECs) and wild-type SCC or with a SCC cell line in which the Dio2 gene (which encodes the D2 protein) was suppressed using CRISPR/Cas9 technology (Santa Cruz, Dallas, Texas, USA, cod. Sc402262) (SCC D2KO) [16] (Figure S2C). We observed that although proliferation ability was higher in HCAEC-D2WT co-culture than in HCAEC alone, HCAEC-D2KO co-culture showed attenuation of this effect on proliferation compared to HCAEC-D2WT co-culture (Figure 1H,I). In addition, the morphology of ECs changed after co-culture with cancer cells. In fact, HCAECs became elongated when co-cultured with SCC WT, but not when co-cultured with HCAEC-D2KO (Figure 1H, right panel). All these changes were associated to enhanced mRNA expression of *VEGF-A* and of its receptor, *VEGFR2*, in the HCAEC-SCC WT cells, whereas *VEGF-A* mRNA expression was lower in the HCAEC-SCC D2KO cells than in HCAEC-SCC WT cells (Figure 1I,J). Finally, the migration ability of HCAECs was enhanced by the conditional medium of SCC WT cells, while SCC D2KO conditional medium failed to stimulate HCAEC migration (Figure S2D,E). Accordingly, TH treatment increased the expression of *VEGF-A* and its receptor *VEGFR2* in endothelial cells, which indicates that TH is a positive regulator of VEGF-A signal acting at the level of both SCC and endothelial cells (Figure S2F,G).

### 3.2. In Vivo D2-Depletion Reduces VEGF-A-Induced Angiogenesis

**Figure 2:**
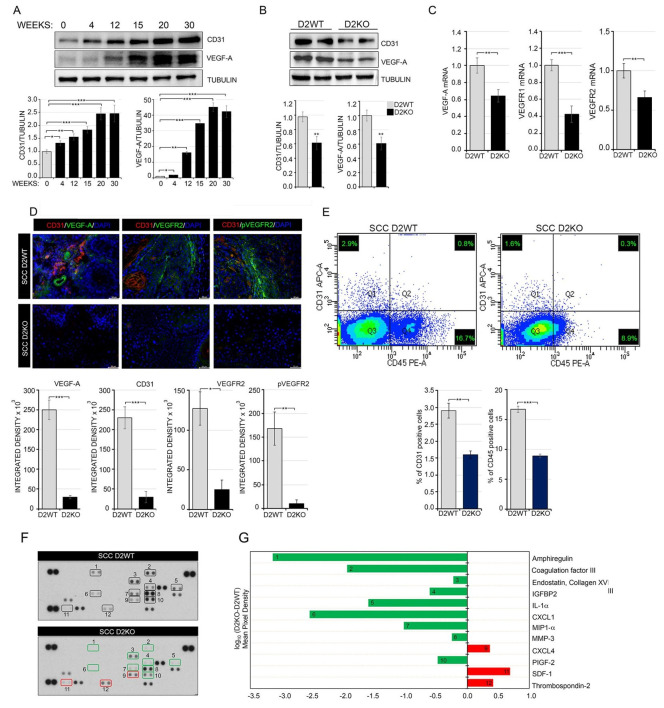
Epidermal attenuation of TH signaling in D2KO mice reduced VEGF-A-induced angiogenesis. (**A**) Western blot analysis of CD31 and VEGF-A expression in SCC skin lesions of mice treated with DMBA-TPA for the indicated times (*n* = 8). Quantification of the single protein versus tubulin levels is represented by diagrams (bottom). (**B**) Western blot analysis of CD31 and VEGF-A expression in skin lesions of D2WT and sD2KO mice (*n* = 15 for both groups) treated with DMBA/TPA for 20 weeks. Quantification of the single protein versus tubulin levels is represented by diagrams (bottom). (**C**) mRNA expression of VEGF-A, VEGFR1 and VEGFR2 in skin lesions of D2WT and D2KO mice (*n* = 15 for both groups). (**D**) CD31, VEGF-A, VEGFR2 and pVEGFR2 immunostaining were performed on paraffin-embedded sections of skin lesions from D2WT and sD2KO mice (*n* = 10 for both groups). Scale bars represent 50 μm. Quantification of single protein levels is represented by histograms (bottom). (**E**) CD31^+^ endothelial population and the CD45^+^ lymphocyte population were analyzed by flow cytometry in SCC lesions of D2WT (*n* = 6) and sD2KO (*n* = 6) mice (bottom). Relative quantification of CD31^+^ and CD45^+^ positive cells. Results are expressed as mean ± SD. * *p* < 0.05, ** *p* < 0.01, *** *p* < 0.001. (**F**) Proteome profiler array of differentially regulated angiogenic proteins in D2WT and sD2KO skin lesions. Duplicate spots: 1, Amphiregulin; 2, Coagulation factor III; 3, Endostatin/Collagen XVIII; 4, IGFBP-2; 5, IL-1α, 6; CXCL1; 7, MIP-1α; 8, MMP-3; 9, CXCL4; 10, PIGF-2; 11, SDF-1; 12, Thrombospondin-2. One representative experiment of 4 replicates is shown. (**G**) Relative protein expression was obtained by comparing the mean pixel density of each factor in sD2KO group with each factor in the D2WT group (log_10_ (D2KO-D2WT)); up-regulated factors are shown in red, down-regulated factors in green.

We next investigated the role of TH-mediated expression of VEGF-A and the endothelial marker CD31 in in vivo SCC tumorigenesis induced by the mouse skin model of two-step chemical carcinogenesis [36]. First, as expected, VEGF-A and CD31 levels increased during SCC tumor progression, which indicates that enhanced tumor vasculogenesis is associated with advanced tumor stages (Figure 2A). To examine further whether TH regulates vessel formation, we analyzed skin lesions from epidermal-specific D2KO mouse models using the K14-CreER; D2^fl/fl^ mice (sD2KO) produced in our lab [16], in which D2 in the epidermal compartment was suppressed by tamoxifen injection. Skin lesions were analyzed 20 weeks after chemical carcinogenesis (Figure S3A). As we previously reported [16], the expression of the proliferative marker K6 is increased in sD2KO mice, whereas K8 expression is reduced, which indicates SCC progression (Figure S3B,C) [16]. Interestingly, VEGF-A and CD31 expression was lower in sD2KO mice than in control mice (D2WT: K14-CreER^−/−^; D2^fl/fl^), (Figure 2B,C). Additionally, immunostaining with VEGF-A antibody revealed lower blood vessel formation in sD2KO skin lesions, which confirms that VEGF-A-induced angiogenesis is significantly suppressed by epidermal D2-depletion (Figure 2D and Figure S4A,B). Notably, the opposite was found in skin lesions from sD3KO mice (in which the TH inactivator enzyme D3 is genetically depleted and thyroid action is enhanced); indeed, VEGF-A and CD31 expression was higher than in D3WT tumors, which confirms that TH boosts tumor angiogenesis (Figure S5A,B). Consistent with the finding of enhanced VEGF-A expression in sD3KO mice, we observed that systemic hyperthyroidism led to increased expression of and consequent angiogenesis (Figure S5C–F).

Next, we analyzed murine ECs lining tumor blood vessels (tumor endothelial cells, TECs) by FACS analysis using antibodies against the EC markers CD31 and CD45. TECs were identified as CD31^+^/CD45^−^ cells, which were derived from SCC skin lesions of D2WT and D2KO mice. We found that the number of TECs was significantly lower in sD2KO lesions than in D2WT lesions (Figure 2E).

To characterize the mechanisms underlying the TH-mediated angiogenic effects, we measured the expression of a panel of angiogenic factors in SCC tumors from sD2KO versus D2WT mice using an angiogenesis array kit. As shown in Figure 2F,G and Figure S3, the pro-angiogenic factors IL-1a, coagulation factor III, CXCL1, and MMP3 were down-regulated in sD2KO tumors, thus confirming that the angiogenic process was reduced in these tumors. Moreover, the anti-angiogenic factors CXCL4, SDF-1 and trombospondin-2 were up-regulated in sD2KO tumors versus D2WT tumors (Figure 2F,G and Figure S6A,B).

### 3.3. Loss of D2 Reduces Tumor Vascularization and Angiogenesis

**Figure 3:**
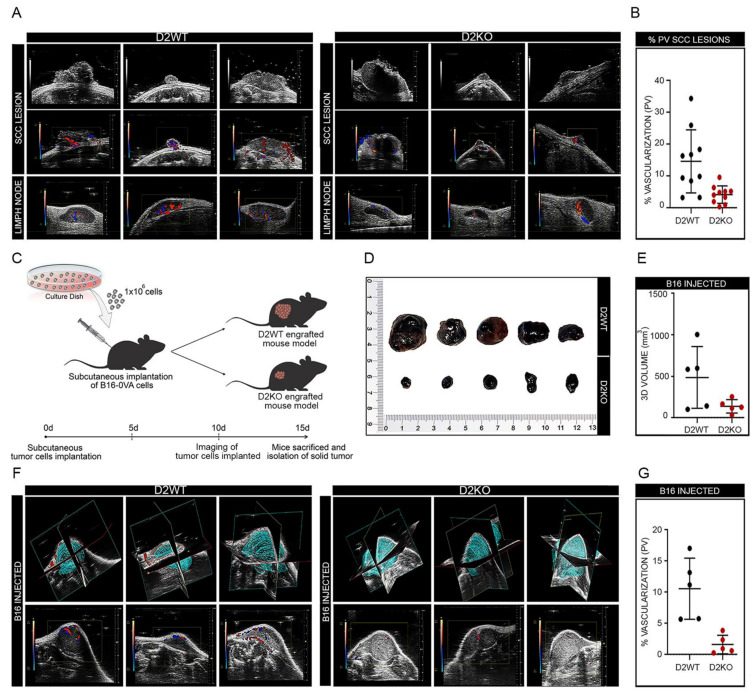
Attenuation of the TH signal drastically affects tumor vascularization and angiogenesis. (**A**) Color/power Doppler of dorsal skin lesions and axillar/inguinal lymph nodes from D2WT (*n* = 10) and sD2KO (*n* = 10) mice treated with DMBA/TPA to evaluate the tumor perfusion. (**B**) Relative quantification of skin lesion vascularization is represented by diagrams. (**C**) Global D2KO mice and D2WT mice were engrafted subcutaneously with 1 × 10^6^ B16-OVA cancer cells (day 0); vascularization and growth of the tumor cells implanted were analyzed. Solid tumors were dissected 15 days after implantation of cancer cells and photographed (**D**); 3D volume (mm^3^) of solid tumors derived from D2WT and D2KO mouse models (**E**) and angiogenesis of solid tumors were evaluated by Color/Power doppler analysis (**F**,**G**).

Tumor angiogenesis was next assessed by color Doppler high-frequency ultrasound analysis of SCC tumors in sD2KO and D2WT mice (*n* = 10). D2WT mice presented SCC tumors with vascular signals around and inside the tumor (Figure 3A). The percentage of vascularization, assessed by the 3D Power Doppler, was, on average, 15% of the total tumoral mass (Figure 3B). Moreover, inguinal and axillar lymph nodes were identified in D2WT mice as homogeneous hypoechoic bean-shaped structures, with contours distinct from surrounding tissues, and the vessel pattern in each lymph node was assessed during color Doppler examination. Conversely, sD2KO mice were characterized by SCC lesions with a much lower vascular signal which was mostly confined around the tumor and was weak inside the tumoral mass, both in the primary SCC lesion and in lymph nodes (Figure 3A). Accordingly, the percentage of vascularization was only 4.2% (Figure 3B). To assess whether as well as being produced by the tumor cells, the T3 derived from the microenvironment sustained angiogenesis, we subcutaneously injected the highly metastatic B16-OVA melanoma cells in wild type and global D2KO mice [37] (Figure 3C). Strikingly, the tumor growth ability was attenuated in D2KO mice versus wild type mice (Figure 3D,E). Moreover, both the 3D Power- and the color Doppler analysis revealed that vascularization was much lower in melanomas grown in D2KO mice than in those grown in wild type mice (Figure 3F,G). Finally, to investigate in greater detail if the loss of D2 and the consequent reduction in the intracellular TH causes reduced vascularization in non-tumoral skin, we performed a color doppler analysis in sD2KO mice 2 weeks after TAM-induced D2 depletion. As shown in Figure S7A,B, the angiogenesis did not differ significantly between sD2KO and WT mice, suggesting that the effects exerted by TH on angiogenesis occur only during tumorigenesis.

### 3.4. Thyroid Hormone Promotes HIF1α Stabilization under Normoxic and Hypoxic Conditions

**Figure 4:**
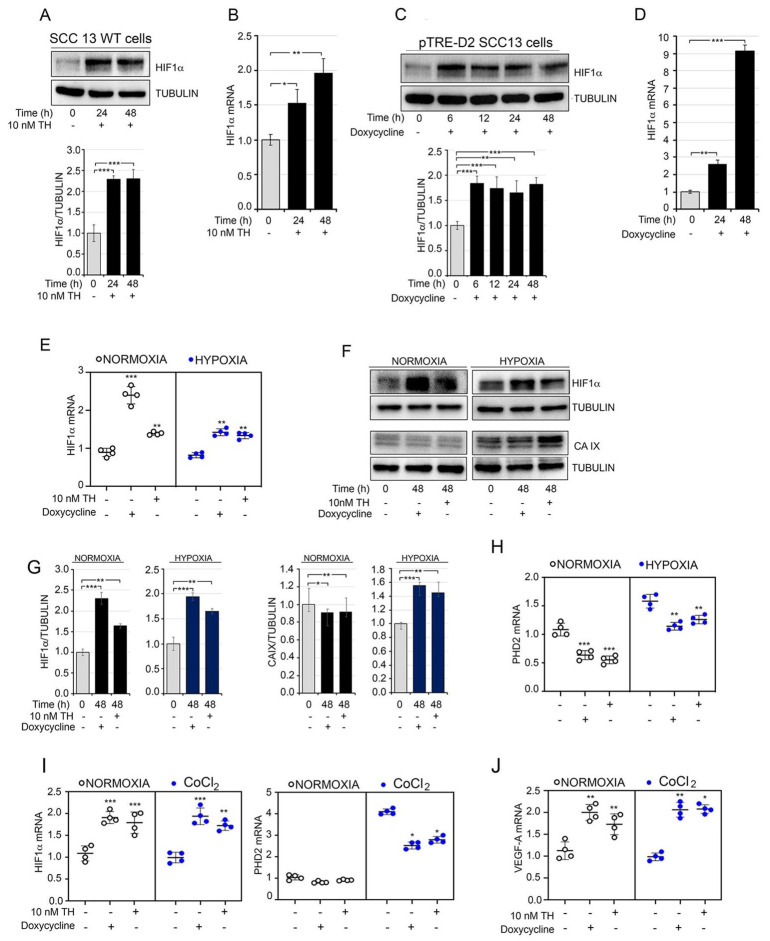
TH regulates the cellular response to hypoxia. (**A**) SCC13 cells were treated with 10 nM TH for 24 and 48 h. Total protein lysates were used for Western blot analysis of HIF1α. Tubulin expression was measured as the loading control (top). Quantification of the HIF1α protein versus tubulin levels is represented by a diagram (bottom). (**B**) mRNA levels of HIF1α in SCC cells treated as indicated in (**A**). (**C**) Western blot analysis of HIF1α was performed in pTRE-D2 SCC13 cells after D2 induction for 6–12–24–48 h. Quantification of the HIF1α protein versus tubulin levels is represented by a diagram (bottom). (**D**) mRNA levels of HIF1α in pTRE-D2 SCC13 cells ±2 μg/mL doxycycline for 24–48 h. * *p* < 0.05, ** *p* < 0.01, *** *p* < 0.001. (**E**) mRNA expression levels of HIF1α under normoxic and hypoxic conditions in pTRE-D2 SCC13 cells after D2 induction or TH treatment for 48 h. (**F**) Western blot analysis of HIF1α and CA IX in pTRE-D2 SCC13 under normoxic and hypoxic conditions after D2 induction or TH treatment for 48 h. (**G**) Quantification of HIF1α and CA IX expression levels are represented by diagrams. (**H**) mRNA levels of PHD2 in pTRE-D2 SCC13 cells under normoxic and hypoxic conditions after D2 induction or TH treatment for 48 h. (**I**,**J**) mRNA levels of HIF1α PHD2 and VEGF-A were measured by real-time PCR analysis in pTRE-D2 SCC13 cells under normoxic and chemical hypoxic conditions (CoCl_2_) after D2 induction or TH treatment for 48 h. One representative experiment of 3 replicates is shown. * *p* < 0.05, ** *p* < 0.01, *** *p* < 0.001.

Enhancement of perfusion through a new vessel network in a tumor microenvironment is often triggered by hypoxic conditions when the expression of VEGF-A is positively regulated by hypoxia-inducible factor α (HIF1α) [38]. Thus, we hypothesized whether TH promotes the activity and accumulation of HIF1α protein in SCCs. We observed that, after 24 and 48 h of treatment, TH induced HIF1α mRNA and protein expression under normoxic conditions (Figure 4A,B); similar results were obtained in pTRE-D2 SCC13 cells following DOX treatment (Figure 4C,D).

To evaluate whether TH plays a role in the cellular response to hypoxia, we performed oxygen deprivation experiments in pTRE-D2 cells after DOX or TH treatment compared to normoxic conditions. We observed that the hyperthyroid conditions (DOX or TH treatment) significantly enhanced the expression of HIF1α (Figure 4E–G). Accordingly, the expression of carbonic anhydrase IX, which is part of the cellular response to oxygen deprivation [27], was enhanced by TH during hypoxia (Figure 4F,G). Interestingly, we also observed that the expression levels of PHD2 (which normally mediates the degradation of HIF1α) were reduced by TH and DOX treatment (Figure 4H), which suggests that TH affects PHD2 and PHD2-mediated HIF1α stability. Similar results were obtained in an experiment of oxygen deprivation induced by cobalt chloride (CoCl_2_) (Figure 4I,J), in which HIF1α was induced by TH in both normoxic and hypoxic conditions.

### 3.5. Thyroid Hormone Accelerates Glycolysis by Driving the Warburg Effect in Squamous Cell Carcinoma

**Figure 5:**
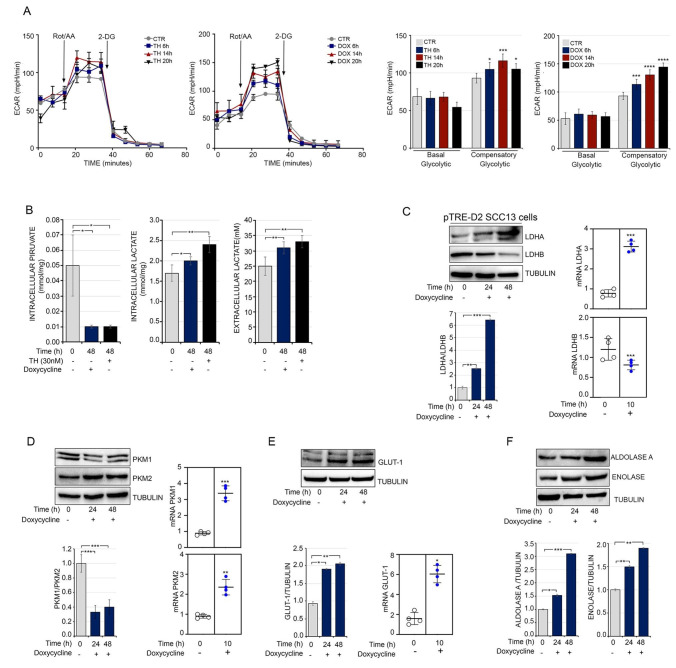
TH drives the metabolic shift in SCC13 cell. (**A**) Assessment of the ECAR showed increased levels of glycolysis in pTRE-D2 SCC13 cells after TH or doxycycline treatment for 6–14–20 h. * *p* < 0.05, ** *p* < 0.01, *** *p* < 0.001, **** *p* < 0.0001. (**B**) pTRE-D2 SCC13 cells were cultured in complete KSFM and treated with TH and doxycycline for 48 h; lactate and pyruvate concentrations were then measured in cell culture supernatant. Each point represents the mean of the duplicates for each sample and each experiment was done in triplicate. * *p* < 0.05, ** *p* < 0.01. (**C**) pTRE-D2 SCC13 cells were treated with doxycycline for 24 and 48 h. Total protein lysates were used for Western blot analysis of LDHA and LDHB. Tubulin expression was measured as loading control (**top**). The LDHA and LDHB ratio is indicated in the diagram (bottom). mRNA levels of LDHA and LDHB in pTRE-D2 SCC13 cells after D2 induction or TH treatment for 10 h (right). (**D**) Western blot analysis of PKM1 and PKM2 was performed in pTRE-D2 SCC13 cells after D2 induction for 24 and 48 h. Tubulin expression was measured as loading control (**top**). The PKM1 and PKM2 ratio is indicated in the diagram (bottom). mRNA levels of PKM1 and PKM2 in pTRE-D2 SCC13 cells after D2 induction or TH treatment for 10 h. (**E**) Expression level of GLUT-1 in pTRE-D2 SCC13 cells after doxycycline treatment by Western blot (**left**) and real-time PCR analysis (right). (**F**) Protein expression level of Aldolase A and Enolase in pTRE-D2 SCC13 cells after doxycycline treatment. Each experiment was performed in triplicates. Results are expressed as the mean ± SD. * *p* < 0.05, ** *p* < 0.01, *** *p* < 0.001.

The above-reported effects of TH and D2 induction on neo-vascularization and response to hypoxia reinforce the concept that TH plays a pro-tumorigenic role. Given that TH is a well-known regulator of cell metabolic bioenergetics, we asked if exogenous TH and the D2-produced intracellular activation contributes to a metabolic shift in SCC cells. We first assessed the ability of TH activation to foster aerobic glycolysis, which is the key trait of cancer cells [39], and conducted bioenergetics profiling of these cells using the Seahorse XF-Bioanalyzer platform. The analysis of glycolytic fluxes indicated that both TH treatment and DOX-induced D2 production promoted glycolytic metabolism, as witnessed by an enhanced extracellular acidification rate (ECAR) (Figure 5A), without any change in total ATP production or oxygen consumption rate (Figure S8A,B). This is consistent with our previous finding that TH induces an Oxidative Phosphorylation (OXPHOS)-to-glycolysis shift in muscle cells [22]. Accordingly, intracellular pyruvate levels were reduced, while both intracellular and extracellular lactate production were increased by TH and D2 (Figure 5B).

To gain better insight into the molecular determinants induced by TH and involved in the increased glycolytic flux, we first assessed whether the expression of lactate dehydrogenase (LDH), which catalyzes the conversion of pyruvate to lactate and is one of the key metabolic enzymes that is altered in cancer [40], is regulated by TH activation. As shown in Figure 5C, we observed a shift from the LDHB isoform to the LDHA isoform, which is typical of cancer cells [40]. Analogously, we observed that the PKM2 isoform, which is known to promote “aerobic-glycolysis”, predominated over its alternative isoform PKM1 upon DOX treatment (Figure 5D). In addition, TH up-regulated the expression of the transmembrane glucose transporter GLUT-1, which suggests that glucose uptake is positively regulated by TH (Figure 5E) and promotes the expression of the glycolytic genes Aldolase A and Enolase (Figure 5F). Considering the close link between neo-angiogenesis, response to hypoxia, and metabolic rewiring of cancer cells [41], we hypothesized whether VEGF-A pharmacological inhibition attenuates the pro-tumorigenic effects of TH. To this aim, EC cells were treated with bevacizumab (a drug composed of an anti-VEGF antibody capable of binding and neutralizing all VEGF isoforms, and largely used for anti-angiogenesis therapy [42]) (Figure S9). Notably, while bevacizumab treatment reduced the expression of Cyclin D1, GLUT-1 and MCT-1, TH reverted such effects, thus showing that *VEGF-A* is not the only TH-target gene in tumor promotion, and that *VEGF-A* is part of pro-tumorigenic cascade in the TH-dependent tumorigenic action.

**Figure 6:**
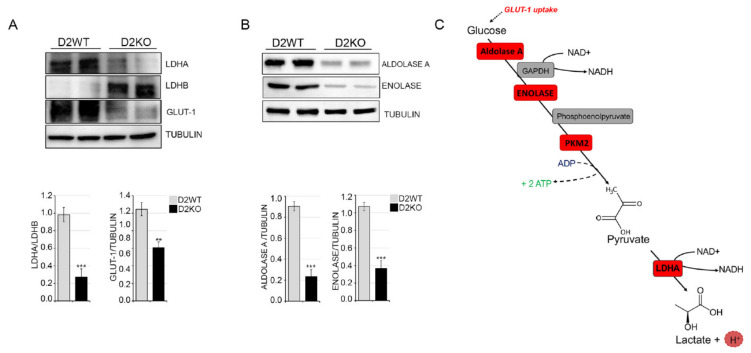
Loss of D2 attenuates tumor metabolic reprogramming. Western blot analysis of LDHA, LDHB and GLUT-1 expression in skin lesions of D2WT and sD2KO mice (*n* = 15 for both groups) treated with DMBA/TPA for 20 weeks. Quantification of the single protein versus tubulin levels is represented by diagrams (bottom). (**B**) The protein expression levels of Aldolase A and Enolase in tissue samples are as indicated in (**A**), and the relative quantifications of the single protein levels are represented by diagrams (bottom). Each experiment was performed in triplicate. Results are expressed as the mean ± SD. ** *p* < 0.01, *** *p* < 0.001. (**C**) Schematic representation of the glycolytic process and the key glycolytic enzymes increased by TH treatment (in red).

**Figure 7:**
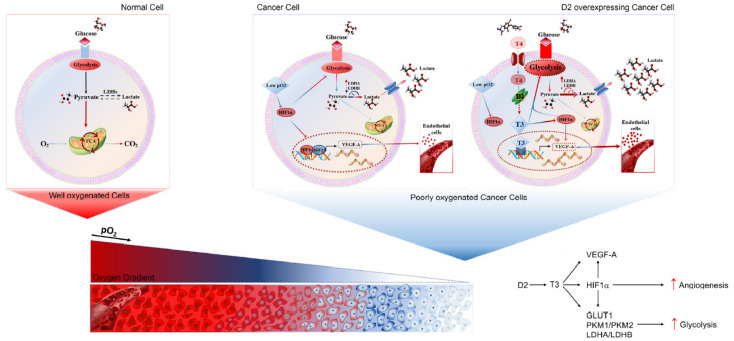
TH exerts a pro-angiogenic and pro-glycolytic function in cancer cells. Graphical representation of the *TH–VEGF-A–HIF1α* regulatory axis in cancer cells during hypoxic conditions.

Finally, given the increase in the glycolytic rate in TH-treated cells, we assessed if the expression levels of glycolytic genes were also differentially regulated in D2KO versus D2WT tumors. Notably, we observed a shift from LDHA to LDHB and down modulation of GLUT-1, Aldolase A and Enolase expression in sD2KO versus D2WT tumors, which is in agreement with the reduced TH status of these tumors (Figure 6A,B). Taken together, these data show that TH induces the Warburg effect and suggest that it plays a hitherto unknown metabolic role by enhancing the expression of key glycolytic enzymes and promoting a global metabolic shift toward aerobic glycolysis (Figure 6C and Figure 7).

## 4. Discussion

Herein, we have reported that TH exerts a pro-angiogenic and pro-glycolytic function in cutaneous SCC tumors. We demonstrated that activation of TH induced by the D2 enzyme is required to foster tumor angiogenesis and vessel formation. Indeed, blockade of D2-mediated TH activation resulted in reduced VEGF-A expression and vascularization of these tumors. Our functional and mechanistic studies indicated that TH induces VEGF-A transcription in SCC cells, thereby resulting in enhanced VEGF-A production and EC proliferation. Contextually, TH augmented the expression of the hypoxia-related genes HIF1α and CA IX, which shows that TH contributes to tumor angiogenesis and to the response to hypoxic conditions. In parallel, we showed that TH is a key driver of metabolic reprogramming of SCC cells. Indeed, upon TH treatment, cancer cells reinforce the key features of aerobic glycolysis such as lactate production, PKM2 and LDHA expression, and increment ECAR, without affecting the oxygen consumption rate.

### 4.1. TH and the Warburg Effect

Tumor cells meet their oxygen need by rewiring metabolic pathways and reprogramming oxidative phosphorylation to glycolysis [39]. Although less energetically efficient than OXPHOS, aerobic glycolysis enables cancer cells to supply carbon sources for the synthesis of nucleic acids, lipids, and amino acids. Moreover, the enhanced glucose uptake enables a high rate of glycolytic fluxes that can meet the energetic requirements of cancer cells. Interestingly, the efficacy of glycolytic metabolism is so potent and successful that rapidly proliferating cancer cells adapt to enhance glycolysis and reduce oxidative phosphorylation, even in the presence of adequate oxygen levels [39].

The established notion that TH fosters glycolytic metabolism in physiological conditions [43,44] suggests that TH-dependent metabolic reprogramming can augment the Warburg effect in cancer. Moreover, TH is a powerful regulator of tumor formation and its invasive progression [11,29], thus adding insights into the potential of TH as a metabolic regulator of cancer biology. Indeed, our results indicate that TH drives the expression of several key glycolytic genes and accordingly fosters glycolytic fluxes. Conversely, the OXPHOS rate, as well as ATP production, remains unchanged.

In line with the classical Warburg effect, we observed that TH induces: (i) the switch from the PKM1 isoform (constitutively activated and expressed in terminally differentiated tissues) to the PKM2 isoform (characteristic of all proliferating cells, especially cancer cells) [45,46]; and (ii) up-regulation of the lactate dehydrogenase gene LDHA. Additionally, this finding is consistent with the pro-tumorigenic effects of TH. Indeed, the LDHA isoform preferentially converts pyruvate to lactate, which is typical of cancer cells, while the LDHB isoform preferentially converts lactate to pyruvate. Enhanced expression of LDHA has been demonstrated to promote the sustained proliferation of cancer cells, and to contribute in promoting the epithelial to mesenchymal transition [47,48,49], angiogenesis [50], cell motility, invasion and migration [51]. Thus, the increased expression of LDHA induced by TH, together with the up-regulation of GLUT-1 and various glycolytic genes, reinforces the concept that TH is a potent tumor-promoting agent.

### 4.2. TH, Angiogenesis and Hypoxia

Crosstalk between hypoxic cancer cells secreting VEGF-A under the control of HIF1α and ECs expressing the VEGF receptor 2 (VEGFR2), triggers EC proliferation and migration, thereby leading to enhanced neo-vascularization [7,52]. VEGF-A gradients increase the motility of ECs, which invade the surrounding extracellular matrix and promote the growth of new vascular sprouts [53]. Our data show that TH is a direct transcriptional regulator of VEGF-A expression in SCC cells, and that TH activation regulates the SCC-EC crosstalk, thereby increasing the proliferation and migration of ECs (Figure 1). As a classical model of cellular crosstalk, we found that TH stimulates *VEGF-A* expression by SCC epithelial cells and the expression of its receptor, *VEGFR1*, by endothelial cells (Figure 1A). Contextually, the reverse effects are promoted by TH, which stimulates VEGF-A and VEGFR2 expression by endothelial cells (Figure S2D,E). These effects exerted by TH on *VEGF-A* expression resemble those observed in muscle cells in which TH-mediated VEGF-A production regulates the muscle stem cell–endothelial cell communication and leads to downstream induction of EC function [34]. Furthermore, these data are consistent with the previous finding that TH promotes angiogenesis in a chick chorioallantoic membrane model and that D2 is expressed in the tumor stroma of colon rectal cancer cells [54,55,56]. Besides inducing *VEGF-A* expression, TH treatment also increases the expression of the receptors *VEGFR2* (in endothelial cells) and *VEGFR1* (in SCC cells). Notably, the TF-binding program predicts 15 putative TREs for *VEGFR1* and 9 TREs for VEGFR2 (data not shown). This suggests that the positive effects of TH on both isoforms can be due to direct regulation by TH.

We also observed that besides inducing *VEGF-A*, TH up-regulates the levels of the HIF1α subunit, which is a key driver of tumor angiogenesis during hypoxia conditions [53]. Although our data show that TH has a direct role in the up-regulation of *VEGF-A* expression, the TH-mediated positive regulation of HIF1α might be either direct or indirect through the down-regulation of PHD2 (Figure 4H). The up-regulation of *VEGF-A* and *HIF1α* can represent a crosslink between the pro-angiogenic and pro-glycolytic effects of TH. Indeed, HIF1α not only promotes neo-vascularization via VEGF-A induction, it also promotes glycolysis to enhance cellular survival under both hypoxic and normoxic conditions [57,58]. Thus, we propose a model by which TH acts a master regulator of glycolytic switch in conditions of low oxygen supply such as in tumor progression states (Figure 7).

To reinforce the concept that TH acts as a potent endocrine switch driving pro-angiogenic and pro-glycolytic effects, we observed that in the presence of VEGF-A pharmacological inhibition by bevacizumab, TH maintains the ability to foster Cyclin D1, GLUT-1 and MCT-1 expression. The latter results indicate that TH is a master regulator of a plethora of genes involved in the promotion of SCC tumorigenesis.

Consistent with this, both vascularization and glycolytic gene expression were reduced in SCC tumors of D2KO mice. This, in turn, suggests that TH regulates aerobic glycolysis either directly via the transcription of glycolytic gene expression, or indirectly mediated by HIF1α.

From a clinical standpoint, our work emphasizes the relevance of TH alteration in cancer progression. Furthermore, the possibility of manipulating TH availability in a precise time- and space-dependent cell context, via deiodinase action, can open the route to novel approaches with which to locally modulate nuclear TH and its pro-tumorigenic action. 

## 5. Conclusions

In summary, our results provide insights into the developing paradigm linking TH signal activation by deiodinase D2 and metabolic reprogramming, transcriptional regulation, and the response of cancer cells to hypoxic conditions. We conclude that a program of metabolic modifications induced by TH acts as a relay, able to interpret the rapid demand in metabolic adaptations of cancer cells during the progression toward invasive transformation.

## Figures and Tables

**Table 1:** Primers used for real-time PCR.

Gene	Forward Primer (5′ → 3′)	Reverse Primer (5′ → 3′)
Cyclophilin A	CGCCACTGTCGCTTTTCG	AACTTTGTCTGCAAACAGCTC
CYCLOPHILIN A	AGTCCATCTATGGGGAGAAATTTG	GCCTCCACAATATTCATGCCTTC
CYCLIN D1	GCTCCTGTGCTGCGAAGTGGA	TCATGGCCAGCGGGAAGACCT
HIF1α	CAGTCGACACAGCCTGGATA	CCACCTCTTTTGGCAAGCAT
PHD2	TGGAGATGGAAGATGTGTGA	GCTCTCTCATCTGCATCAAA
Vegf-a	GCACATAGGAGAGATGAGCTTCC	CTCCGCTCTGAACAAGGCT
VEGF-A	GCACATAGGAGAGATGAGCTTCC	CTCCGCTCTGAGCAAGGCC
Vegfr-1	GCACATGACGGAAGGAAGAC	TTCGCAGTTCAGCAGTCCTA
VEGFR-2	ACAAGTGCTTCTACCGGGAA	GGACCCGAGACATGGAATCA
Vegfr-2	TTGTTGGCGATGAACTCACC	TCCATAGGCGAGATCAAGGC
GLUT-1	ATCCTGCCCACCACGCTCAC	CACGAAGGCCAGCAGGTTCA
MCT-1	TCGGGTGGCTCAGCTCCGTA	AGATACCTGAAATAATTAGG
Keratin 6	GGCTGAGGAGCGGCGTGAACAG	AAGGAGGCAAACTTGTTGTTGAG
Keratin 8	ACAACAAGTTCGCCTCCTTC	TCTCCATCTCTGTACGCTTGT
LDHA	CAACATGGCAGCCTTTTCCT	ACCCACCCATGACAGCTTAA
LDHB	GCGTGATTGGAAGTGGATGT	AACACCTGCCACATTCACAC
PKM1	CAGCCAAAGGGGACTATCCT	GAGGCTCGCACAAGTTCTTC
PKM2	CTATCCTCTGGAGGCTGTGC	GTGGGGTCGCTGGTAATG
VEGFA-TRE-ChIP	CCCAAAAGCAGGTCACTCAC	CGGCTTGGGGAGATTGCTCTA

## Supplementary Materials

The following are available online at https://www.mdpi.com/article/10.3390/cancers13112743/s1, Figure S1: Conditional Dio2 expression in SCC13 cells, Figure S2: Alteration of TH signaling affects the proliferation and migration of endothelial cells, Figure S3: Effective D2-depletion in the epidermal compartment in SCC mouse model, Figure S4: D2 ablation strongly affects tumor vascularization and angiogenesis, Figure S5: D3 ablation or TH treatment leads to enhanced tumor angiogenesis, Figure S6: TH orchestrates angiogenesis-related protein expression [59,60,61,62,63,64,65,66,67,68,69,70,71], Figure S7: Loss of D2 does not affect vascularization in normal skin, Figure S8:TH activation does not change ATP production or oxygen consumption, Figure S9: Inhibition of VEGF-A signal in HCAECs is partially rescued by TH, Figure S10: Full western blots.
