# Supplementary material for: Thyroid Hormone Enhances Angiogenesis and the Warburg Effect in Squamous Cell Carcinomas
Source: Cancers (Basel). 2021 Jun 1;13(11):2743. doi: 10.3390/cancers13112743 (PMC8199095; doi:10.3390/cancers13112743)
Supplement: Supplementary file 1 [file cancers-13-02743-s001.zip › cancers-1195985-supplementary/cancers-1195985 Figure S10 original images.pdf]

Figure 1A

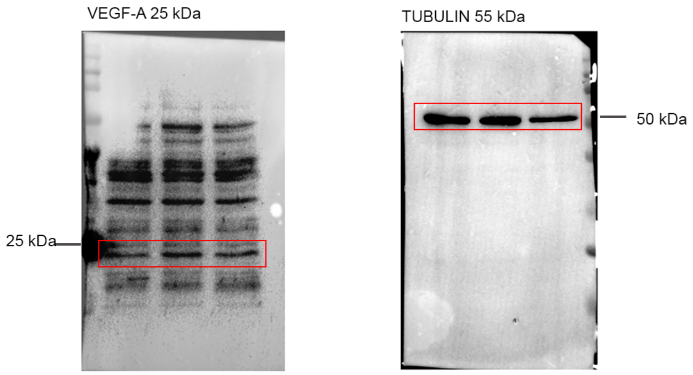

Figure 1C

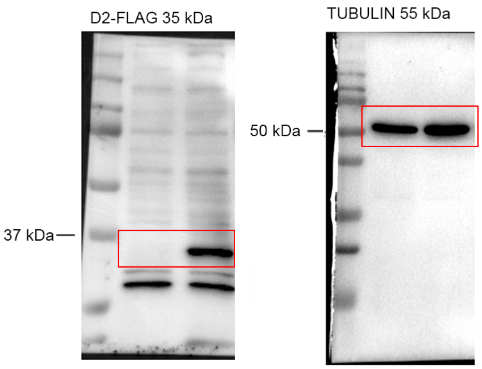

Figure 1D

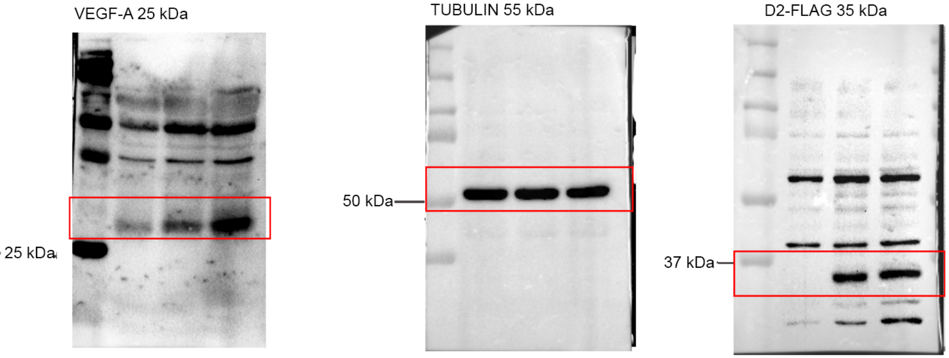

Figure 2A

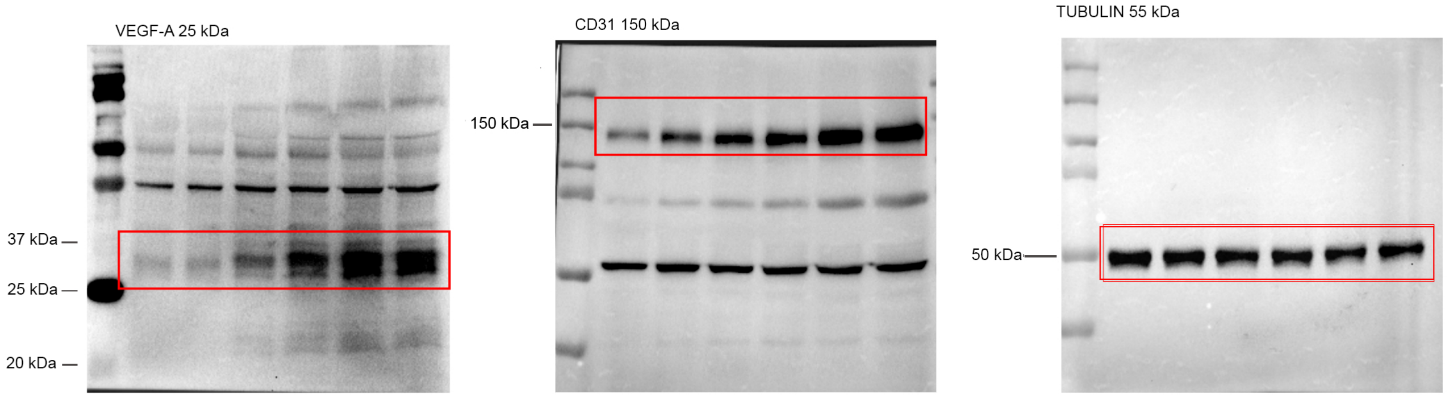

Figure 2B

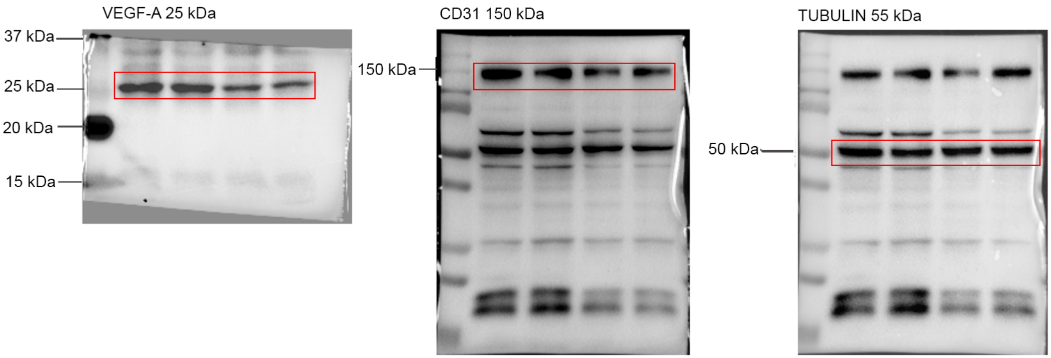

Figure 4A

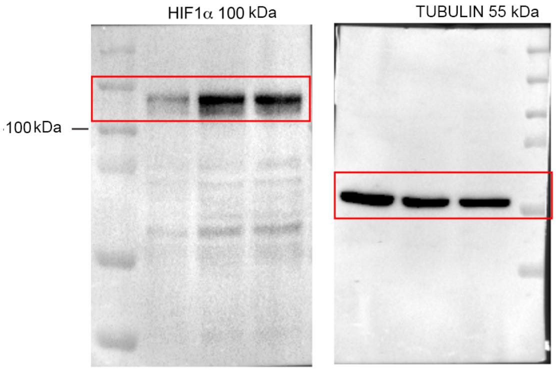

Figure 4C

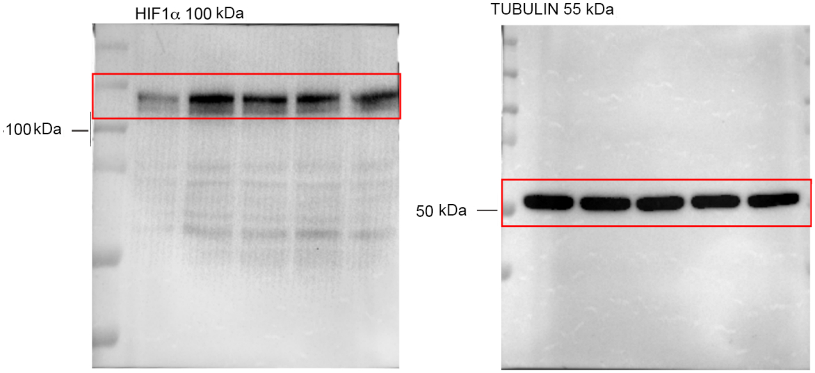

Figure 4F

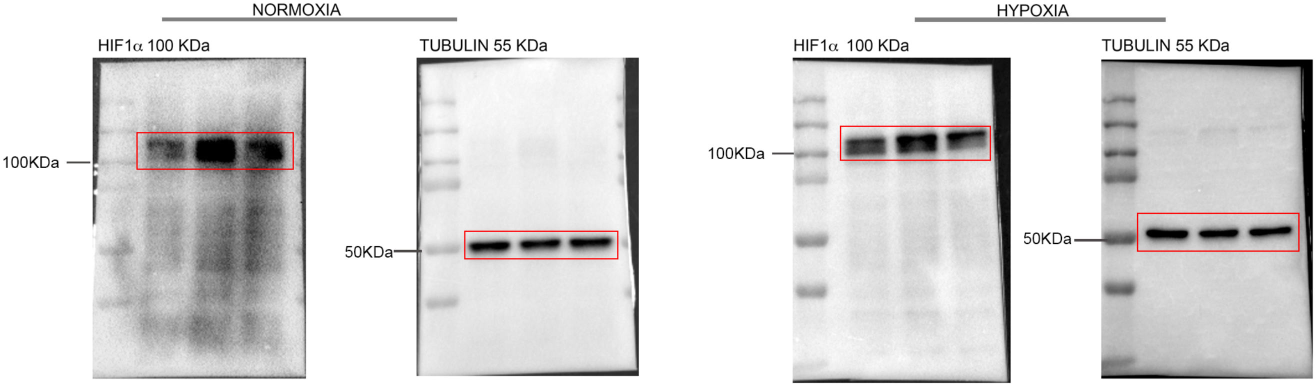

Figure 4F

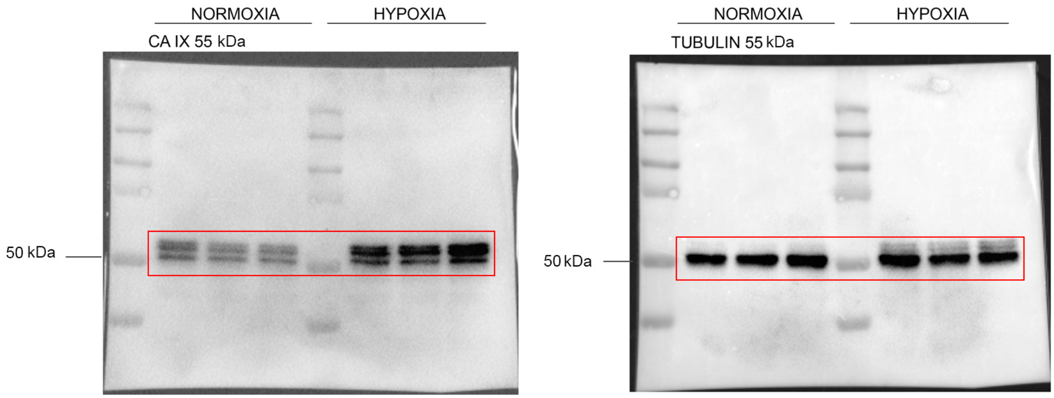

Figure 5C

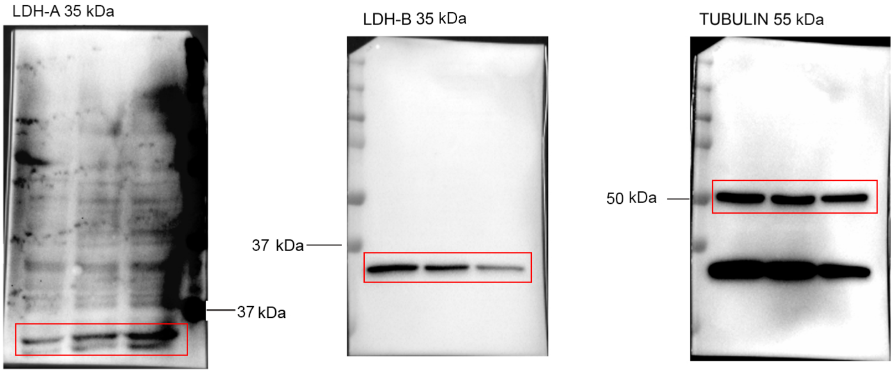

Figure 5D

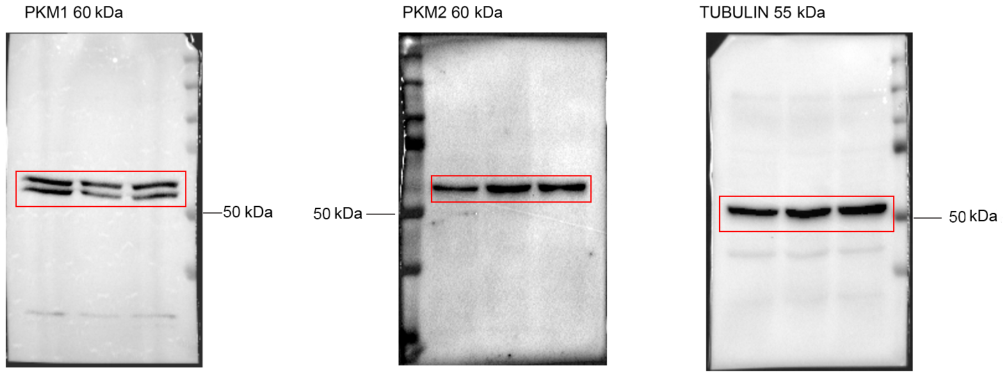

Figure 5E

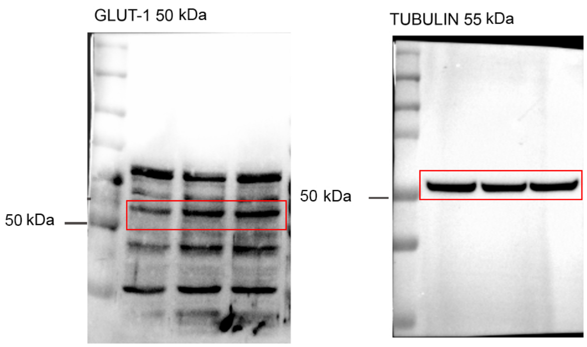

Figure 5F

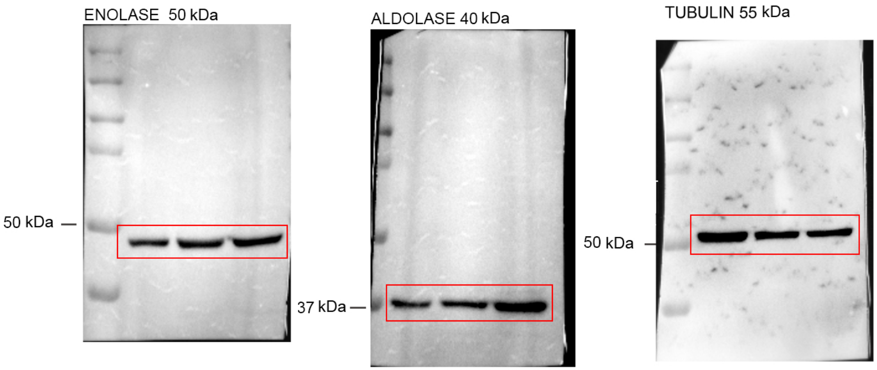

Figure 6A

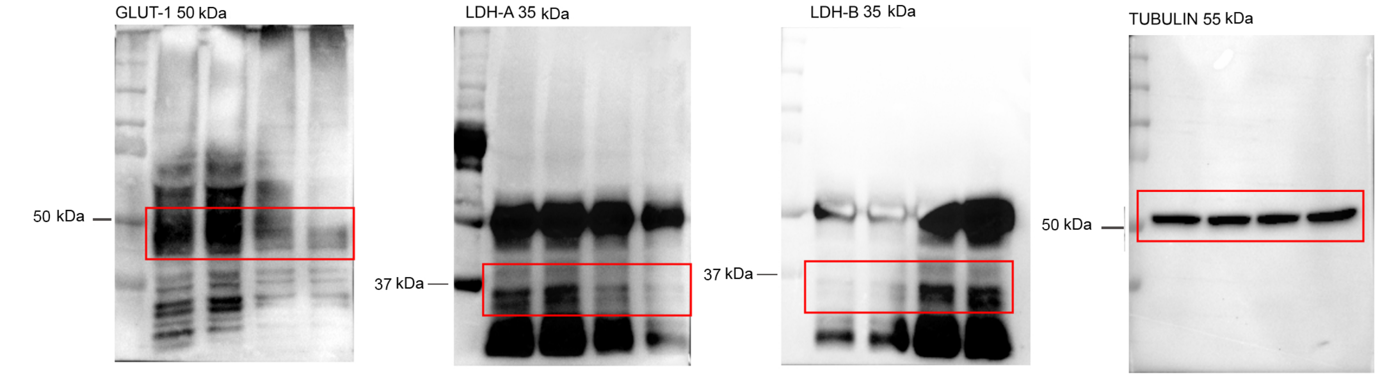

Figure 6B

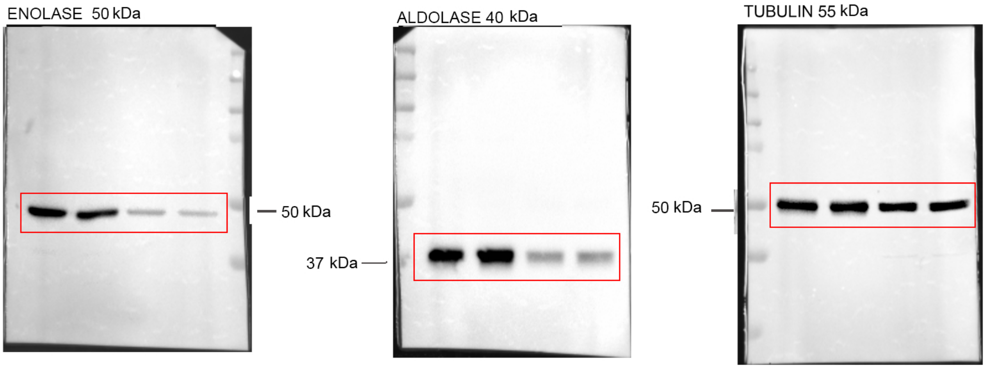

Figure S1

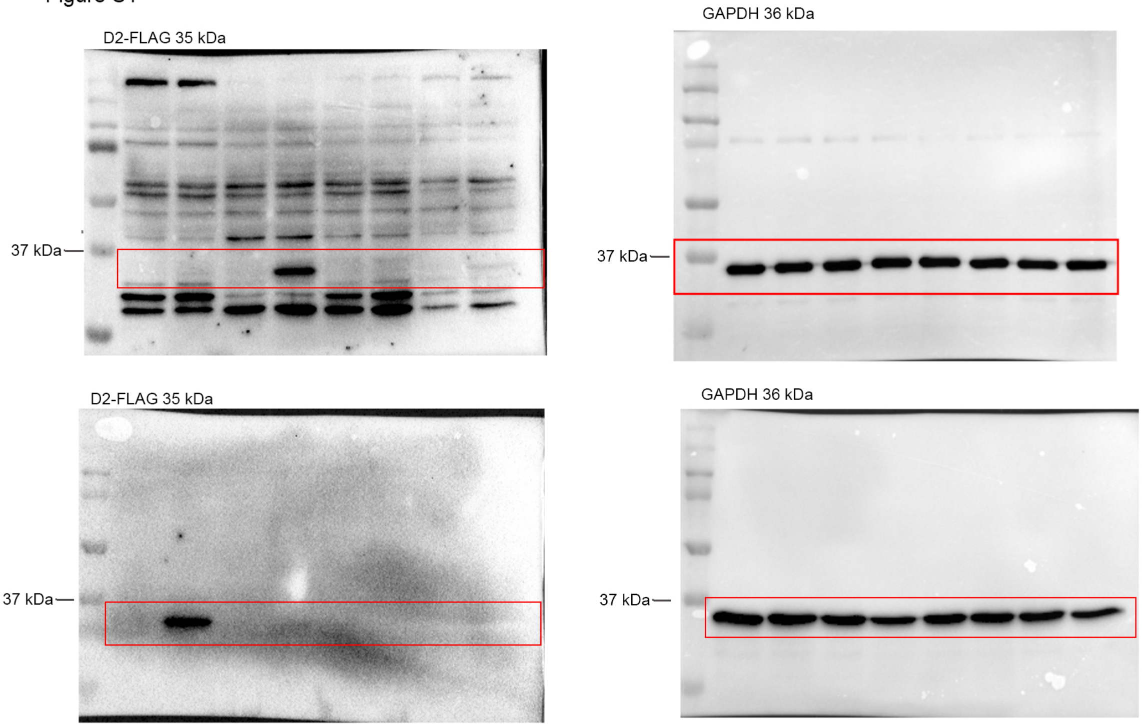
D2-FLAG 35 kDa
37 kDa
GAPDH 36 kDa
37 kDa
D2-FLAG 35 kDa
37 kDa
GAPDH 36 kDa
37 kDa
