# Supplementary material for: Thyroid Hormone Enhances Angiogenesis and the Warburg Effect in Squamous Cell Carcinomas
Source: Cancers (Basel). 2021 Jun 1;13(11):2743. doi: 10.3390/cancers13112743 (PMC8199095; doi:10.3390/cancers13112743)
Supplement: Supplementary file 1 [file cancers-13-02743-s001.zip › cancers-1195985-supplementary/cancers-1195985-supplementary-done by RAE.pdf]

cancers

MDPI

Supplementary Materials

Thyroid Hormone Enhances Angiogenesis and the Warburg Effect in Squamous Cell Carcinomas

Caterina Miro, Annarita Nappi, Annunziata Gaetana Cicatiello, Emery Di Cicco, Serena Sagliocchi, Melania Murolo, Valentina Belli, Teresa Troiani, Sandra Albanese, Sara Amiranda, Ann Marie Zavacki, Mariano Stornaiuolo, Marcello Mancini, Domenico Salvatore and Monica Dentice

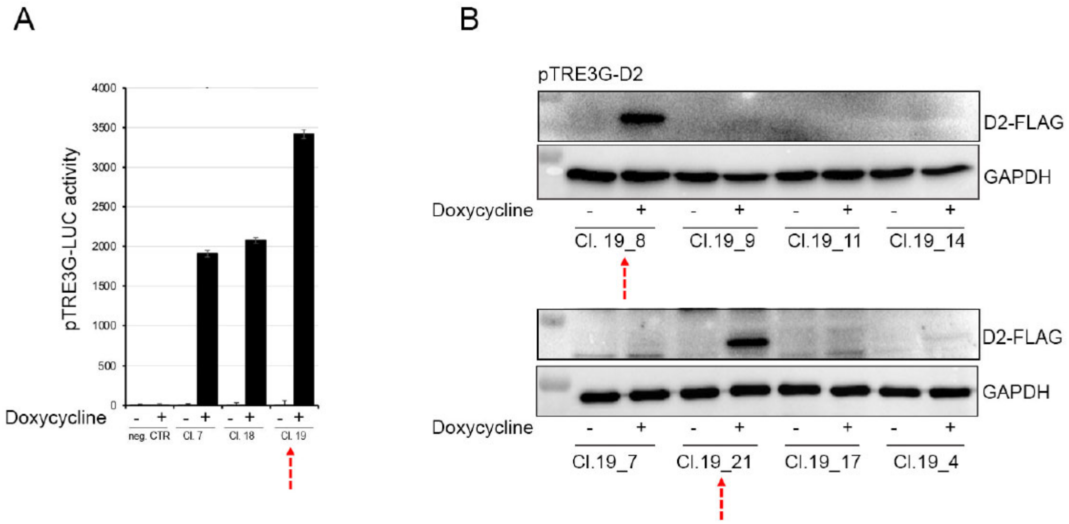

Figure S1. Conditional Dio2 expression in SCC13 cells. (**A**) SCC13 cells were stably transfected with the pCMV-Tet3G plasmid. After isolation of G418-resistant clones (**A**), stable transfection was verified by a second transfection of positive clones with the pTRE3G-Luc and CMV-Renilla as internal control. The results are shown as means of the luciferase/renilla (LUC/Renilla) ratios. Among all, three clones revealed high luciferase induction as indicated in the diagram (clone 7, clone 18, clone 19). (**B**) The expression level of D2 in clones transfected with the pTRE3G-D2 plasmid, after doxycycline treatment, was evaluated by western blot analysis using an anti-Flag M2 antibody (D2-Flag). Two clones with the highest fold induction of the D2 (clone 19_21 and 19_8) were selected for propagation and further testing. GAPDH expression was measured as loading control.

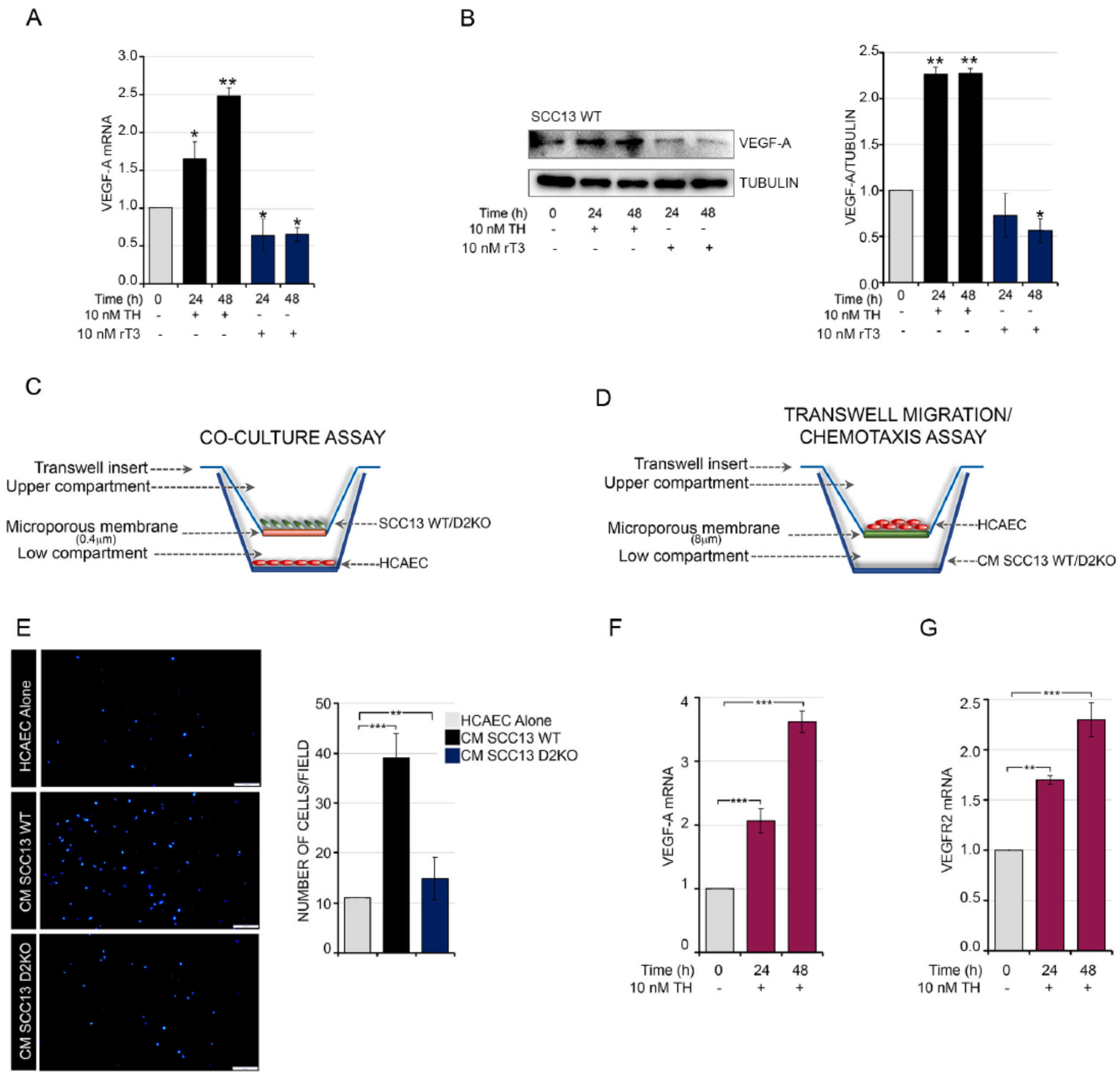

Figure S2. Alteration of TH signaling affects the proliferation and migration of endothelial cells. (**A**) VEGF-A mRNA expression in SCC13 cells treated with 10 nM TH or rT3 for 24 and 48 h. (**B**) Western blot analysis of VEGF-A was performed in SCC13 cells as indicated in (**A**). Quantification of the VEGF-A protein versus tubulin levels is represented by a diagram. (**C**) Schematic diagram of co-culture of HCAEC and SCC13WT or D2KO cells. Abbreviations: HCAEC, human coronary artery endothelial cells; SCC13, human epithelial squamous cell carcinoma. (**D**) Schematic diagram of transwell migration assay of HCAEC cultured in the upper compartment and conditioned medium of SCC13WT or D2KO cells in the lower compartment. Abbreviations: CM, conditioned medium. (**E**) HCAEC migration through a transwell insert towards a lower chamber containing WT- or D2KO-CM was determined. Cells were stained with DAPI live cell stain. (**F**,**G**) mRNA expression of VEGF-A and VEGFR2 was measured in HCAEC cells treated with 10 nM TH for 24 and 48 h. Each experiment was done in triplicate. Results are expressed as mean ± SD. * $p < 0.05$, ** $p < 0.01$, *** $p < 0.001$.

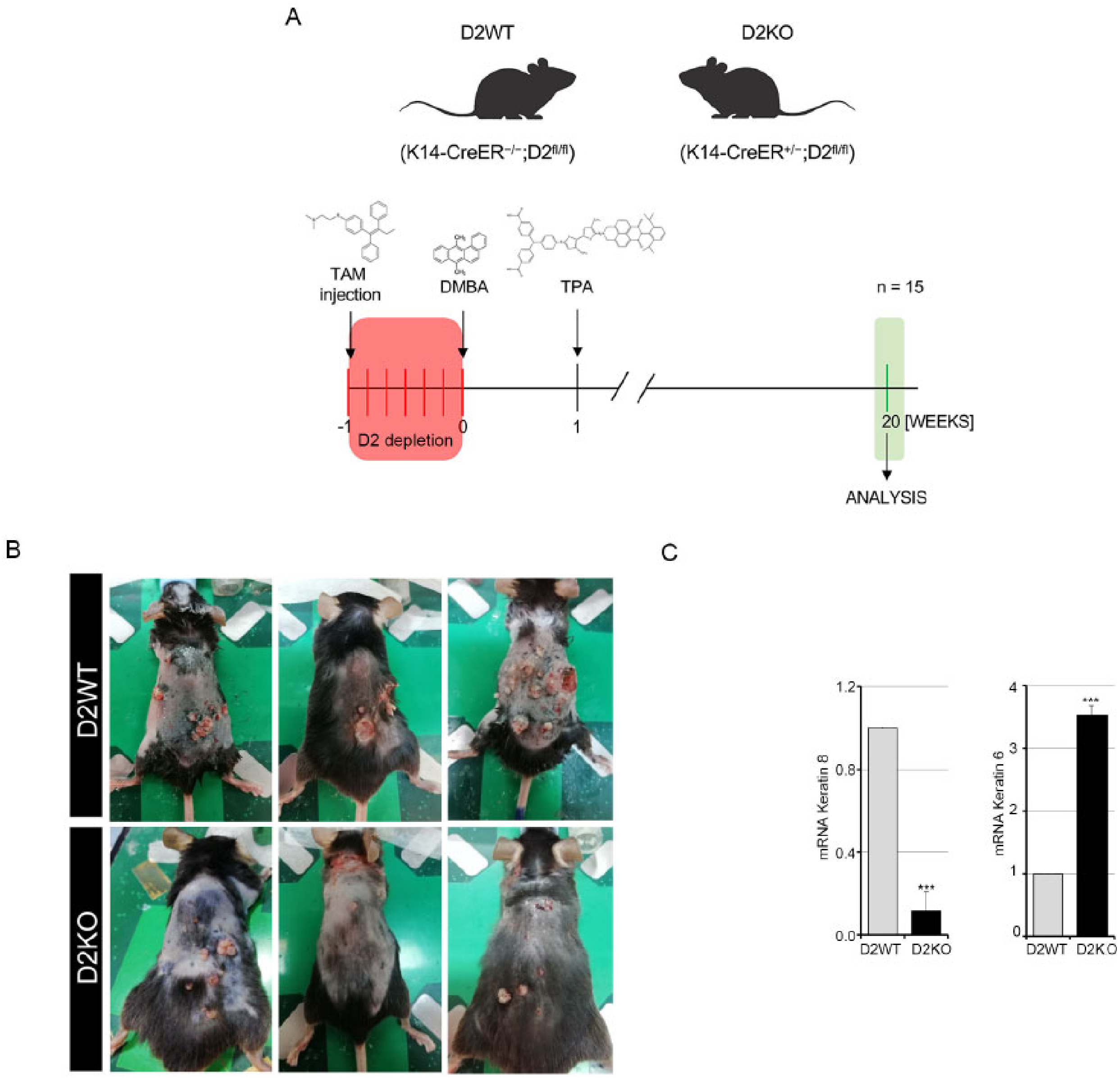

Figure S3. Effective D2-depletion in the epidermal compartment in SCC mouse model. (**A**) Schematic representation of D2-depletion and the two-step carcinogenesis experiment in a total number of 15 D2WT and 15 sD2KO mice ($n = 15$). (**B**) Picture of the dorsal back skin from D2WT ($n = 15$) and sD2KO ($n = 15$) mice treated with DMBA/TPA for 20 weeks. (**C**) mRNA expression of Keratin 8 and Keratin 6 in skin lesions of D2WT and sD2KO mice ($n = 15$ for both groups). Results are expressed as mean ± SD. *** $p < 0.001$.

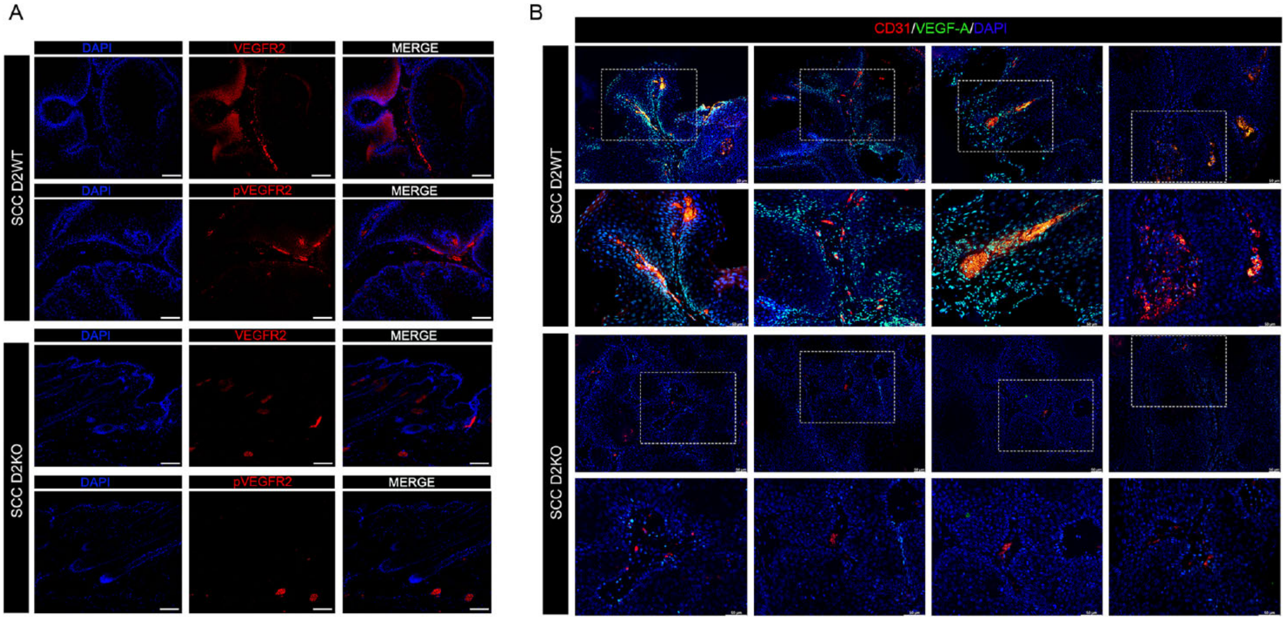

Figure S4. D2 ablation strongly affects tumor vascularization and angiogenesis. (**A**) Immunostaining for VEGFR2 and pVEGFR2 was performed on paraffin-embedded sections of dorsal back skin lesions from sD2KO and D2WT mice (n = 10 for both groups). Scale bars represent 50 µm**.** (**B**) CD31 and VEGF-A immunostaining was performed on paraffin-embedded sections of skin lesions from D2WT and sD2KO mice (n = 10 for both groups). Scale bars represent 50 µm.

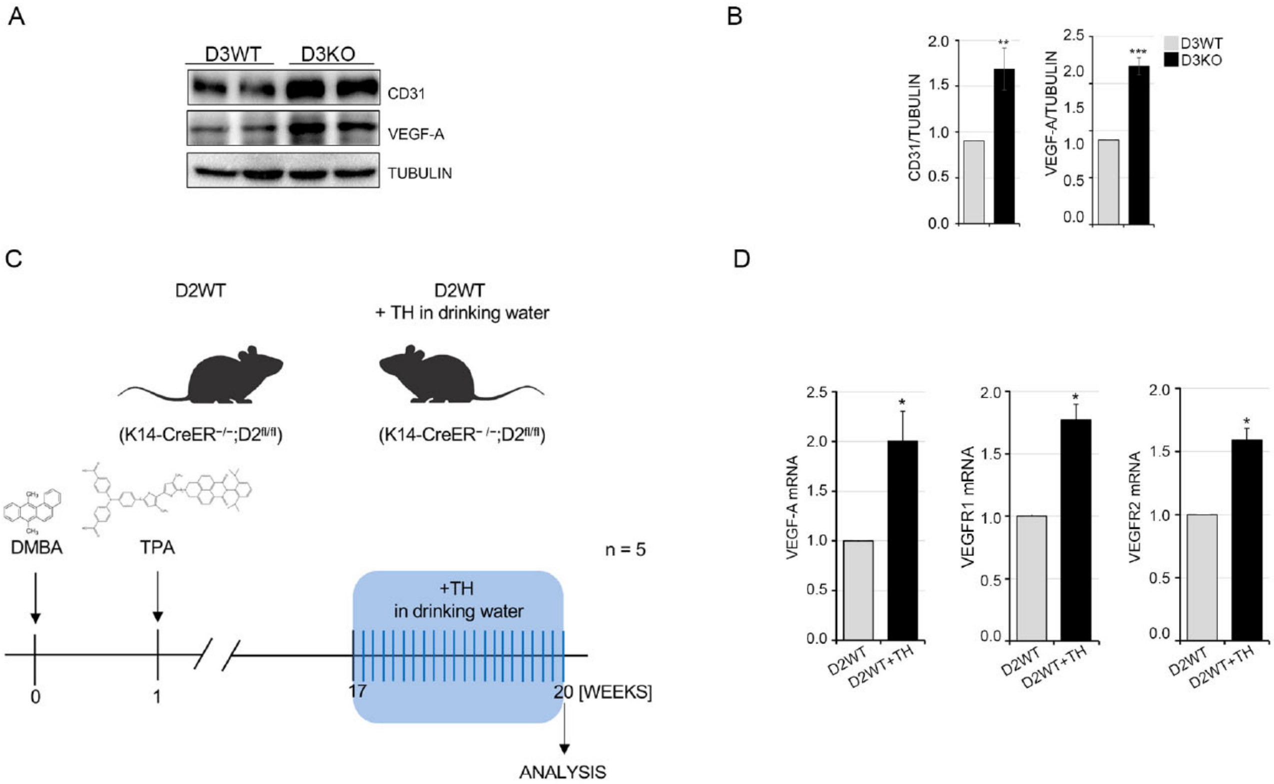

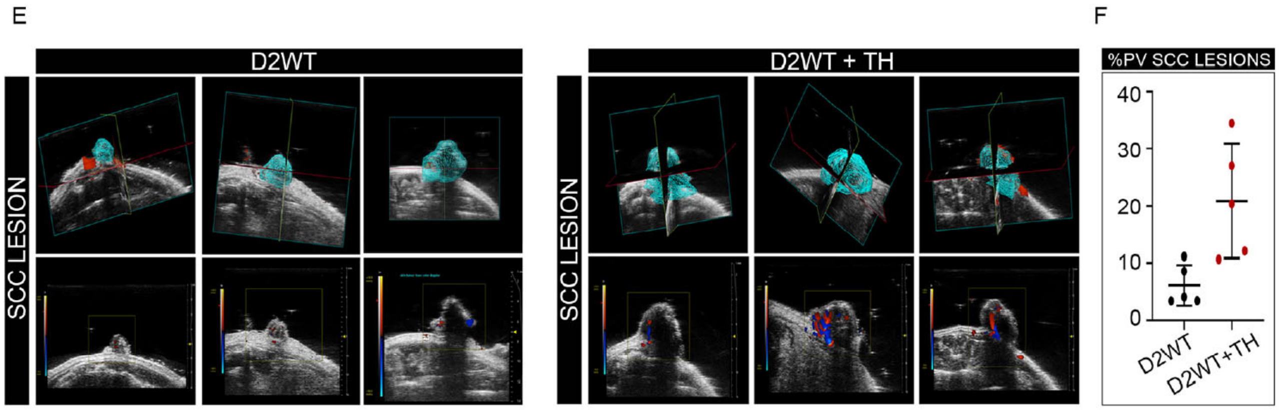

Figure S5. D3 ablation or TH treatment leads to enhanced tumor angiogenesis. (**A**) Western blot analysis of CD31 and VEGF-A expression in skin lesions of D3WT (*n* = 15) and sD3KO (*n* = 15) mice treated with DMBA/TPA for 20 weeks. (**B**) Quantification of CD31 and VEGF-A expression levels (A) versus tubulin is represented by diagrams. (**C**) Schematic representation of the two-step carcinogenesis experiment in a total number of 10 mice treated with DMBA/TPA for 17 weeks, among these 5 were treated with T3 (1 mg/mL) and T4 (4 mg/mL) in drinking water for 3 weeks to obtain hyperthyroid mice. (**D**) Real time PCR analysis of VEGF-A, VEGFR1 and VEGFR2 mRNA expression in skin lesions from D2WT and hyperthyroid mice (indicated as D2WT + TH) treated with DMBA/TPA for 17 weeks (*n* = 5 for both groups). Results are expressed as mean ± SD. *$p < 0.05$. (**E**) Color/power Doppler of dorsal skin lesions from D2WT (*n* = 5) and hyperthyroid (*n* = 5) mice treated with DMBA/TPA to evaluate the tumor perfusion. (**F**) Relative quantification of skin lesion vascularization is represented by diagrams.

A

ANALYTE	ALTERNATIVE NOMENCLATURE	FUNCTION	MEAN PIXEL DENSITY (CTR)	MEAN PIXEL DENSITY (D2KO)	REFERENCES
Amphiregulin	AR	Pro-angiogenic action	2563005	1866.5	[59]
Coagulation Factor III	Tissue Factor, TF	Pro-angiogenic action	1868257.5	21547.5	[60]
Endostatin/Collagen XVIII	___	Endogenous inhibitor of angiogenesis	2941170	1751212.5	[61]
IGFBP-2	___	Pro-angiogenic action	867000	214710	[62, 63]
IL1α	IL-1F1	Pro-angiogenic action	1709392.5	43332.5	[64]
KC	CXCL1	Pro-angiogenic action	962497.5	2748.5	[65]
MIP-1α	CCL3	Pro-angiogenic action	1682872.5	159120	[66]
MMP-3	___	Pro-angiogenic action	4293435	2426070	[67]
Platelet Factor 4	CXCL4, PF4	anti-angiogenic action	677917.5	1570545	[68]
PlGF-2	___	Pro-angiogenic action	2679285	889842.5	[69]
SDF-1	CXCL12	anti-angiogenic action	21037.5	103020	[70]
Thrombospondin-2	TSP-2	anti-angiogenic action	1007760	2636062.5	[71]

B

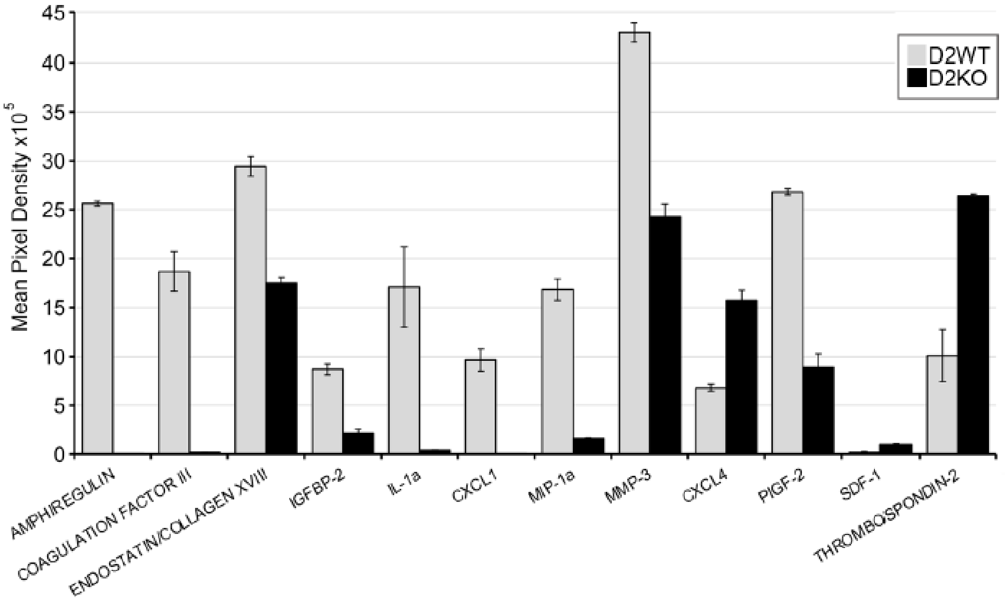

Figure S6. TH orchestrates angiogenesis-related protein expression. (**A**) Proteome profiler array of differentially regulated angiogenic proteins with an angiogenesis antibody array in D2WT and sD2KO tissue samples. One representative experiment of 4 is shown. (**B**) Quantification of relative protein expression by comparing the mean pixel density of each factor in sD2KO group with that of each factor in the D2WT group was indicated by diagram.

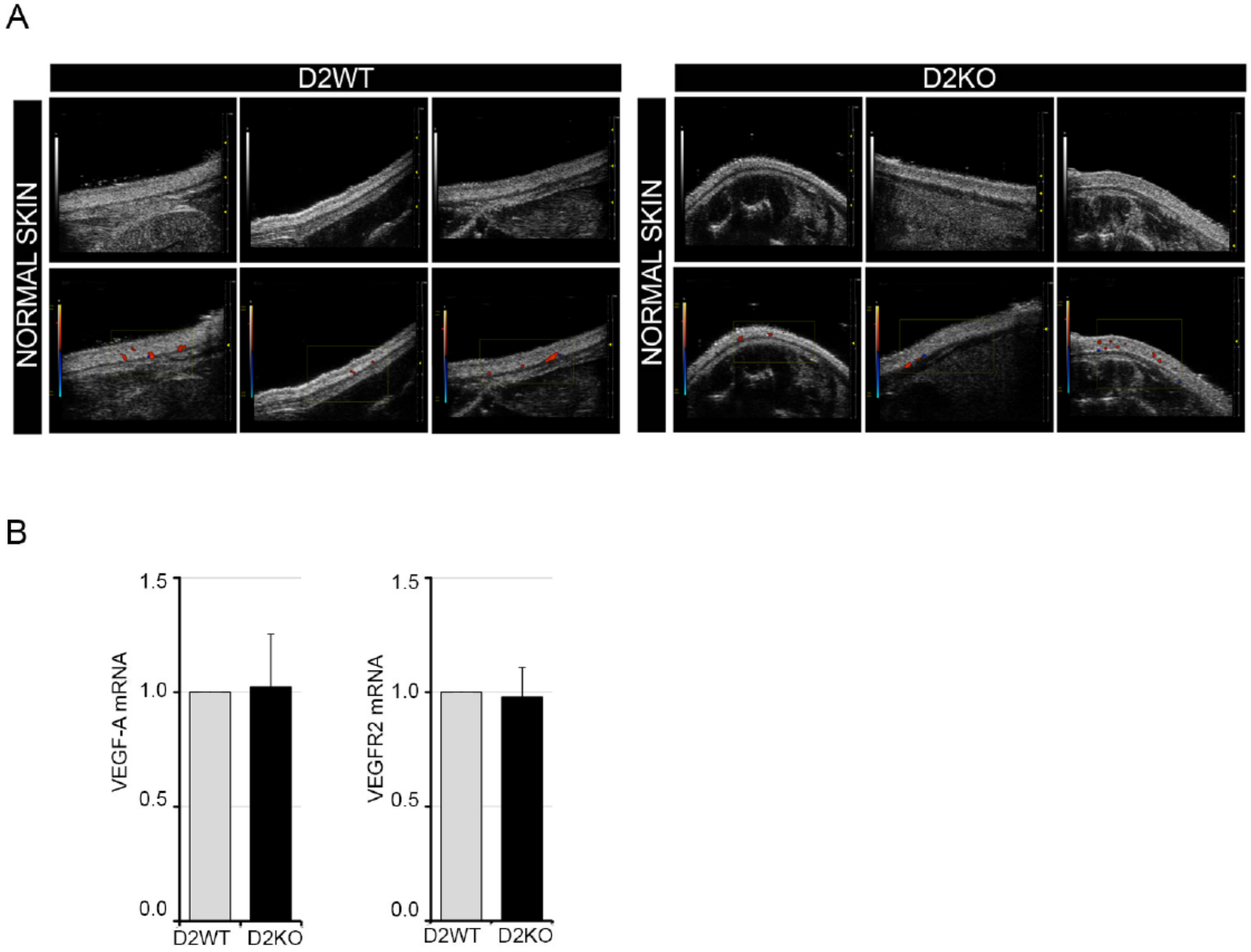

Figure S7. TH attenuation in sD2KO mice is not associated to reduced vasculogenesis in normal skin. Color/power Doppler analysis of dorsal skin from D2WT ($n = 5$) and sD2KO ($n = 5$) mice both injected with TAM as already described two weeks before analysis. (**B**) Real time PCR analysis of VEGF-A and VEGFR2 mRNA expression in dorsal skin from D2WT ($n = 5$) and sD2KO ($n = 5$) mice.

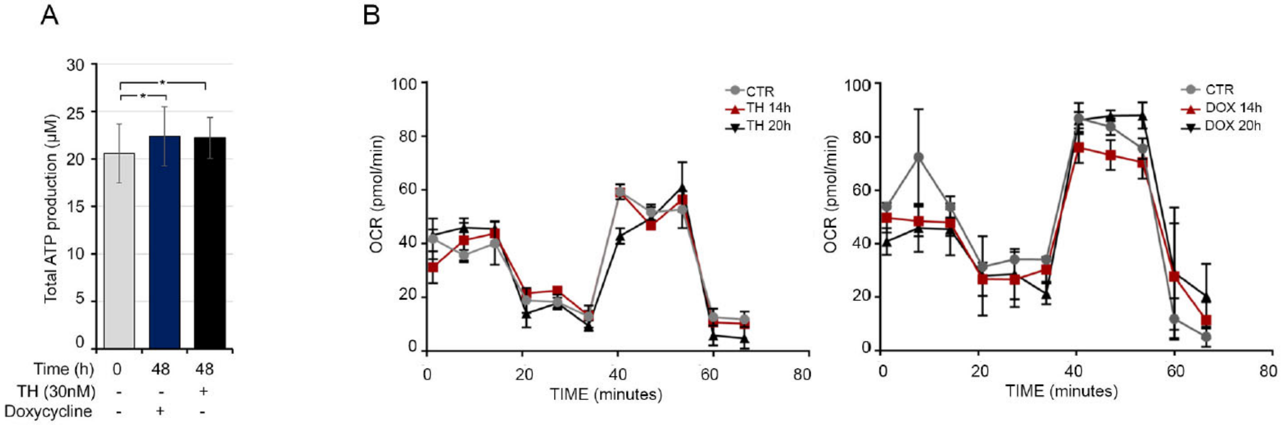

Figure S8. TH activation does not change ATP production or oxygen consumption. (**A**) ATP production was measured in pTRE-D2 SCC13 cells treated with doxycycline for 48 hours using the ATPlite kit. (**B**) pTRE-D2 SCC13 cells were treated with TH (10 nM) or doxycycline for 14 and 20 h.

A

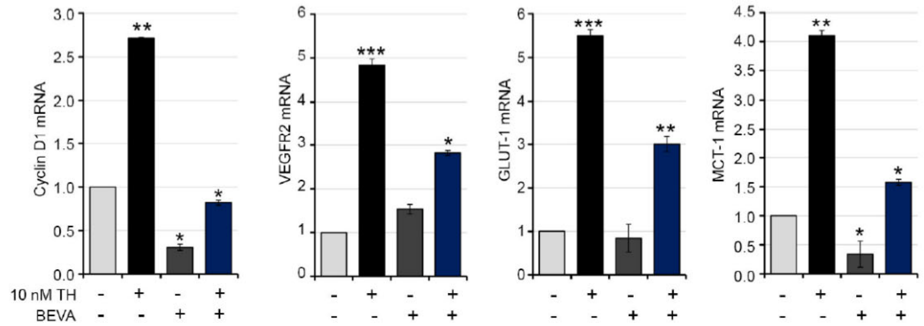

Figure S9. Inhibition of VEGF-A signal in HCAECs is partially rescued by TH. (**A**) Real time PCR analysis of Cyclin D1, VEGFR2, GLUT-1 and MCT-1 in HCAEC cells treated with 10 nM TH and Bevacizumab (BEVA, 500 ng/mL) for 24 h. Results are expressed as mean ± SD. * $p < 0.05$, ** $p < 0.01$, *** $p < 0.001$.
